# Transcriptional Reprogramming of Rice Cells by *Xanthomonas oryzae* TALEs

**DOI:** 10.3389/fpls.2019.00162

**Published:** 2019-02-25

**Authors:** Stefanie Mücke, Maik Reschke, Annett Erkes, Claudia-Alice Schwietzer, Sebastian Becker, Jana Streubel, Richard D. Morgan, Geoffrey G. Wilson, Jan Grau, Jens Boch

**Affiliations:** ^1^Department of Plant Biotechnology, Institute of Plant Genetics, Leibniz Universität Hannover, Hanover, Germany; ^2^Institute of Computer Science, Martin Luther University Halle-Wittenberg, Halle, Germany; ^3^New England Biolabs Inc., Ipswich, MA, United States

**Keywords:** TALE, *Xanthomonas oryzae*, rice, salicylic acid, virulence, plant pathogen, type III effector, genome

## Abstract

Rice-pathogenic *Xanthomonas oryzae* bacteria cause severe harvest loss and challenge a stable food supply. The pathogen virulence relies strongly on bacterial TALE (transcription activator-like effector) proteins that function as transcriptional activators inside the plant cell. To understand the plant targets of TALEs, we determined the genome sequences of the Indian *X. oryzae* pv. *oryzae* (*Xoo*) type strain ICMP 3125^T^ and the strain PXO142 from the Philippines. Their complete TALE repertoire was analyzed and genome-wide TALE targets in rice were characterized. Integrating computational target predictions and rice transcriptomics data, we were able to verify 12 specifically induced target rice genes. The TALEs of the *Xoo* strains were reconstructed and expressed in a TALE-free *Xoo* strain to attribute specific induced genes to individual TALEs. Using reporter assays, we could show that individual TALEs act directly on their target promoters. In particular, we show that TALE classes assigned by AnnoTALE reflect common target genes, and that TALE classes of *Xoo* and the related pathogen *X. oryzae* pv. *oryzicola* share more common target genes than previously believed. Taken together, we establish a detailed picture of TALE-induced plant processes that significantly expands our understanding of *X. oryzae* virulence strategies and will facilitate the development of novel resistances to overcome this important rice disease.

## Introduction

With more than half of the world’s population consuming rice as a staple food, an understanding of the molecular basis of pathogen virulence systems is urgently needed to develop resistant plants and secure a stable food supply. Bacterial leaf blight is the most serious bacterial disease of rice with harvest losses up to 50%. It is caused by the Gram-negative bacterium *Xanthomonas oryzae* pv. *oryzae* (*Xoo*) (Liu et al., 2014). The related bacterial pathogen *Xanthomonas oryzae* pv. *oryzicola* (*Xoc*) does not cause as severe symptoms with harvest losses up to 32% but is currently emerging as an important global rice disease (Liu et al., 2014). The major difference between both pathovars is their mode of infection. While *Xoc* enters the plant through stomata or wounds and infects the parenchyma tissue, *Xoo* invades through hydathodes and wounds to colonize the xylem and spread rapidly via the vascular system (Ou, 1985; Noda and Kaku, 1999; Niño-Liu et al., 2006; White and Yang, 2009).

Both *Xoo* and *Xoc*, rely on the type-III-secretion system-dependent translocation of a plethora of effector proteins into the cytoplasm of plant cells (White and Yang, 2009). Among these effectors are transcription activator-like effectors (TALEs), which activate gene expression of host genes to support the infection (Boch and Bonas, 2010). Once TALEs are inside the host plant cell, they are transported into the cell nucleus, bind to target promoter regions and induce the expression of target plant genes (Van den Ackerveken et al., 1996; Gu et al., 2005; Kay et al., 2007). The target sequence is bound by the central repeat region of a TALE, which contains up to 33.5 repeats. Each repeat is typically 34 amino acids long and recognizes one base in the DNA in a sequential fashion (Boch et al., 2009; Moscou and Bogdanove, 2009). Two hypervariable residues, termed “repeat variable diresidue” (RVD), at position 12 and 13 control the base specificity of each repeat (Boch et al., 2009; Moscou and Bogdanove, 2009). After binding their target sequence, TALEs presumably recruit the transcription initiation complex by interacting with the transcription initiation factor IIA α and γ subunits (Yuan et al., 2016; Huang et al., 2017; Ma et al., 2018). A C-terminal acidic activation domain in TALEs is needed to efficiently initiate transcription (Van den Ackerveken et al., 1996; Zhu et al., 1998) in an area approximately 40–60 bp downstream of their binding region, but the exact transcription start site depends on the relative position of the TALE to other promoter elements (Hummel et al., 2012; Streubel et al., 2017).

*Xoo* and *Xoc* strains can carry significant numbers of *TALE* genes, depending on the geographic origin of the bacterial strain: up to 10 TALEs for African *Xoo*, 19 TALEs for Asian *Xoo*, 28 TALEs for Asian *Xoc* and no TALEs for North-American *Xoo* (Gonzalez et al., 2007; Triplett et al., 2011; Booher et al., 2015; Grau et al., 2016; Quibod et al., 2016). So far, several TALE-induced target genes have been identified that support virulence of the pathogen. The best studied TALE targets are the clade III *SWEET* genes, which efficiently support growth of *Xoo* (Streubel et al., 2013). SWEET proteins are sugar exporters which presumably provide nutrients for the pathogen (Chen et al., 2010, 2012; Zhou et al., 2015). To date, three different *SWEET* genes are TALE targets in rice. *OsSWEET11* is induced by the TALE PthXo1 (also known as TalBX1), *OsSWEET13* is addressed by PthXo2 (TalAM2) and *OsSWEET14* is targeted by PthXo3 (TalBH1), TalC (TalBS1), AvrXa7 (TalAC6) and Tal5 (Yang and White, 2004; Yang et al., 2006; Antony et al., 2010; Römer et al., 2010; Yu et al., 2011; Streubel et al., 2013; Zhou et al., 2015). TALE-mediated *SWEET* gene induction has also been described for the interaction of *Xanthomonas* with cotton and cassava (Cohn et al., 2014; Cox et al., 2017). This indicates that sugar export is a central virulence hub for *Xanthomonas* infections of different plants.

Another major group of virulence targets are transcription factors. In rice, the bZIP transcription factor *OsTFX1* is targeted by PthXo6 (TalAR) from Asian *Xoo* strains, whereas *OsTFX1* and the IXc AP2/ERF transcription factor *OsERF#123* are both targeted by TalB from African *Xoo* strains (Sugio et al., 2007; Tran et al., 2018). In pepper, the bHLH transcription factor *UPA20* is induced by AvrBs3 from *Xanthomonas campestris* pv. *vesicatoria* causing a hypertrophy of leaf cells (Kay et al., 2007). In citrus, *CsLOB1* is induced by PthA4, PthA^W^ and PthA^∗^ from *X. citri* pv. *citri* and by PthB and PthC from *X. citri* pv. *aurantifolii* causing hyperplasia and rupture of the epidermis in infected tissue (Al-Saadi et al., 2007; Hu et al., 2014; Li et al., 2014). In tomato, a bHLH transcription factor, induced by AvrHah1 from *X. gardneri* upregulates the expression of a pectate lyase responsible for formation of water soaking symptoms (Schwartz et al., 2017). In contrast to this reorganization of plant-expressed genes, induction of the general transcription initiation factor *OsTFIIAγ1* by PthXo7 is a specific means for *Xoo* to overcome a point mutation in rice that results in resistance. The *xa5* mutation of the gene *OsTFIIAγ5* in the rice variety IRBB5 disrupts the ability of TALEs to interact with this basal transcription factor (Iyer and McCouch, 2004; Huang et al., 2016; Yuan et al., 2016). By expressing the paralog *OsTFIIAγ1*, PthXo7 can restore normal TALE function (Sugio et al., 2007). For *Xoc* only very few TALE virulence targets have been described. The putative sulfate transporter *OsSULTR3;6*, is targeted by Tal2g and is involved in lesion expansion and bacterial exudation of *Xoc* (Cernadas et al., 2014). In addition, there are two proposed TALE targets induced by *Xoo* and *Xoc* alike, the RNA methyltransferase OsHEN1 and the putative flavone synthase type I OsFNS (also described as F3H), but no effect on the bacterial infection could be shown for either one (Cernadas et al., 2014).

TALEs can also trigger plant resistance. In rice, the NLR (nucleotide-binding domain, leucine-rich repeat) protein Xa1 present in rice variety IRBB1 recognizes full-length TALEs (Ji et al., 2016). All sequenced Asian *Xoo* and *Xoc* strains contain truncated TALE genes with premature stop codons and deletions in their N-terminal region, which render them incapable of gene induction. These TALE variants suppress the *Xa1*- and *Xo1-*dependent recognition of TALEs and were termed iTALEs (interfering TALEs) or truncTALEs (truncated TALEs), accordingly (Ji et al., 2016; Read et al., 2016). In addition, the induction of so-called executor resistance genes by TALEs can also trigger a resistance reaction (Gu et al., 2005; Liu et al., 2007; Wu et al., 2008; Tian et al., 2014; Wang et al., 2015).

To help decipher TALE functions, the AnnoTALE prediction software and TALE nomenclature has been established that is based on the similarity of the repeat regions and the DNA-binding specificity of TALEs (Grau et al., 2016).

In order to understand TALE diversity, evolution, and the virulence strategies of *X. oryzae* pv. *oryzae* (*Xoo*), knowledge about the complete TALE repertoire (TALomes) of *Xoo* strains from different origins is essential. At present, 14 different Asian *Xoo* strains originating in Korea, Japan, Taiwan, and the Philippines (11 strains) have been fully sequenced (Table 1) (Ochiai et al., 2005; Salzberg et al., 2008; Streubel et al., 2013; Booher et al., 2015; Quibod et al., 2016; Char et al., 2018; NZ_CP011532.1). Nevertheless, detailed information about *Xoo* strains from the important rice growing country of India is lacking. Although draft genomes for Indian *Xoo* strains have been published, data on their *TALE* genes is not available because the highly repetitive nature of TALEs precludes a correct assembly of short Illumina sequencing reads. ICMP 3125^T^ is the type strain of *Xoo* and thus widely available and frequently used. A draft genome of the type strain without assembled *TALE* genes has been available since 2013 (under the strain number ATCC 35933, PRJNA195863).

**Table 1:** General features of completely sequenced *Xoo* strains.

Strain^1^	Race	TALE genes	Genome size (Mbp)	GC content (%)	PacBio coverage	Sampling region; country (year)	Reference
**ICMP 3125^T^**	–	17	4.99	63.7	170×	West Godavari; India (1965)	This study
KACC 10331	–	13	4.94	63.7	–	Korea	Lee et al., 2005
MAFF 311018	–	17	4.94	63.7	–	Japan	Ochiai et al., 2005
PXO71	4	19	4.91	63.7	102×	Palawan; Philippines (1974)	Quibod et al., 2016
PXO83	2	18	5.03	63.7	170×	Nueva Ecija; Philippines (1976)	Grau et al., 2016
PXO86	2	18	5.02	63.7	200×	Laguna; Philippines (1977)	Booher et al., 2015
PXO99^A^	6	19	5.24	63.6	200×	Laguna; Philippines (1980)	Salzberg et al., 2008; Booher et al., 2015
**PXO142**	3	19	4.99	63.7	376×	Davao; Philippines (1981)	This study
PXO145	7	18	5.04	63.7	121×	Mountain Province; Philippines (1982)	Quibod et al., 2016
PXO211	8	17	5.03	63.7	183×	Ifugao; Philippines (1989)	Quibod et al., 2016
PXO236	5	16	4.97	63.7	146×	Ifugao; Philippines (1989)	Quibod et al., 2016
PXO282	1	15	4.96	63.7	268×	Nueva Vizcaya; Philippines (1990)	Quibod et al., 2016
PXO524	9b	17	4.95	63.7	152×	Laguna; Philippines (1994)	Quibod et al., 2016
PXO563	10	18	4.94	63.7	173×	Laguna; Philippines (1998)	Quibod et al., 2016
PXO602	3c	20	4.95	63.7	191×	Quezon; Philippines (2006)	Quibod et al., 2016
XF89b	–	17	4.97	63.7	–	Taichung; Taiwan (1987)	NZ_CP011532.1


*^*1*^*Xanthomonas oryzae* type strain is indicated by a superscript T and newly sequenced strains are shown in bold.*

In this study, we present a detailed overview of *Xoo* TALE targets and provide evidence for functional convergence between TALEs of *Xoo* and *Xoc* strains. We sequenced the two *Xoo* strains PXO142 and ICMP 3125^T^ from the Philippines and India, respectively, to evaluate the TALE repertoires of strains from diverse geographic locations. The TALEs were grouped into 23 TALE classes, and a new system to categorize TALEs depending on their frequency in sequenced *Xoo* strains is proposed. *In planta* targets of TALEs were identified using RNAseq of infected rice tissue in combination with *in silico* target prediction. Twelve TALE targets could be assigned to specific TALEs. Among these targets, three have previously been published, five have been hypothesized to be targets, and four are new targets.

## Materials and Methods

### Bacterial Growth Conditions

*Escherichia coli* strain Top10 (New England Biolabs, Frankfurt am Main, Germany) was grown in LB medium at 37°C, *Agrobacterium tumefaciens* strain GV3101 was grown in YEB medium at 28°C and *Xanthomonas oryzae* pv. *oryzae* (*Xoo*) strains PXO83, PXO142, ICMP 3125^T^ and Roth X1-8 were cultivated in PSA medium at 28°C.

### Plant Growth Conditions and Inoculation

*Oryza sativa* ssp. *japonica* cv. Nipponbare was grown under glasshouse conditions at 28°C (day) and 25°C (night) at 70% relative humidity (RH). Leaves of 4-week-old plants were infiltrated with a needleless syringe and a bacterial suspension as previously described (Reimers and Leach, 1991). *Nicotiana benthamiana* plants were grown under 16 h of light, 40–60% RH, at 23°C (day) 19°C (night) in a growth chamber. Leaves of 4- to 6-week-old plants were inoculated with *A. tumefaciens* strains using a needleless syringe.

### Genome Assembly

Libraries of genomic DNA of *Xoo* strains ICMP 3125^T^ and PXO142 were sequenced on a Pacific Biosciences RS II instrument. The library for *Xoo* strain ICMP 3125^T^ was sequenced using two SMRT cells yielding a total of 68,513 reads with an N50 read length of 22,914 bp. Reads were assembled using the HGAP_Assembly.2 pipeline from the Pacific Biosciences SMRT Portal with default parameters. This resulted in two contigs, one of 12,940 bp with only 35× coverage and one large contig of approximately 5 Mbp. Due to spurious coverage, the first contig was removed. The latter contig could be circularized manually and was shifted such that *dnaA* was located at position 45 in forward orientation relative to the origin. After circularization and shifting, this contig was further refined in an additional polishing step using the Resequencing.1 pipeline from the Pacific Biosciences SMRT Portal, yielding a chromosome of 4,990,672 bp in total. In resequencing, coverage across the chromosome was largely uniform (Supplementary Figure S1) with an average coverage of 170×.

The library for *Xoo* strain PXO142 was also sequenced using two SMRT cells with a total of 166,038 reads and an N50 read length of 23,191 bp. For PXO142, the HGAP_Assembly.2 pipeline was executed using default parameters except for “Minimum Subread Length = 3000” and “Minimum Seed Read Length = 5000,” yielding a single contig of approximately 5 Mbp. This contig was also further polished by the Resequencing.1 pipeline and could be circularized using the Circlator pipeline (Hunt et al., 2015) with default parameters, resulting in a chromosome of 4,982,118 bp in total. Finally, this chromosome was also shifted such that dnaA was located at position 45 in forward orientation relative to the origin. Again, coverage was largely uniform with an average coverage of 376×, except for a coverage peak at approximately 45 kbp that did not overlap any TALE genes (Supplementary Figure S2). Genome sequences of *Xanthomonas* strains have been deposited in NCBI GenBank^1^ under accessions CP031697 (ICMP 3125^T^) and CP031698 (PXO142).

### TALE Prediction

From the genomes of *Xoo* ICMP 3125^T^ and *Xoo* PXO142, respectively, *TALE* genes were predicted by the “TALE Prediction” tool of AnnoTALE (Grau et al., 2016) (version 1.3) and subsequently assigned to TALE classes by the “TALE class assignment” tool. For *Xoo* ICMP 3125^T^, 17 *TALE* genes (including two pseudo genes) were predicted, two of which belong to novel TALE classes TalES and TalET. For *Xoo* PXO142, 19 *TALE* genes (including three pseudo genes) were predicted, which were all assigned to classes already present in the AnnoTALE class builder.

### Prediction of TALE Target Genes

TALE target genes and corresponding target boxes were predicted by the “Predict and Intersect Targets” tools of AnnoTALE (Grau et al., 2016). To this end, putative promoter sequences were extracted from the rice genome (MSU7 genome and annotation^2^^,^^3^ ) as sequences spanning from 300 bp upstream of the transcription start site (TSS) until 200 bp downstream of the TSS or the start codon, whichever comes first, as proposed previously (Grau et al., 2013).

### RNA-Seq

Rice cultivar Nipponbare leaves were inoculated with *Xoo* strains PXO142, ICMP 3125^T^, or 10 mM MgCl_2_ as a mock control in five spots in an area of approximately 5 cm using a needleless syringe. Two leaves of three rice plants each were inoculated for each strain and control, respectively. 24 h later, samples were taken, frozen in liquid nitrogen, and RNA prepared. Three replicates of this experiment were done on separate days and subjected to RNAseq analysis, separately. Stranded libraries were sequenced on an Illumina HiSeq 2500 instrument (Eurofins Genomics) as 100 bp single-end reads. General statistics of the sequencing data are provided in Supplementary Table S1.

RNA-seq data after inoculation with *Xoo* ICMP 3125^T^ and *Xoo* PXO142 were adapter clipped using cutadapt (v1.15) (Martin, 2011) and quality trimmed using trimmomatic (v0.33) (Bolger et al., 2014) with parameters “SLIDINGWINDOW:4:28 MINLEN:50.” Transcript abundances were computed by kallisto (Bray et al., 2016) using parameters “–single -b 10 -l 200 -s 40” using the cDNA sequences^4^ as reference transcripts. Differentially expressed genes relative to the control were determined by the R-package sleuth (Pimentel et al., 2017). Since replicates have been paired during library preparation and sequencing, the replicate was considered as an additional factor when computing *p*-values of differential expression. Differential expression was aggregated on the level of genes using the parameter target_mapping of the sleuth function sleuth_prep(), and log_2_-fold change and Benjamini–Hochberg-corrected *P*-value were recorded. Gene abundances and sleuth outputs with regard to differential expression are provided as Supplementary Data S1.

RNA-seq reads were also mapped to the rice genome (MSU7) to obtain detailed information about transcript coverage and transcription starts. To this end, adapter clipped and quality trimmed reads were mapped using TopHat2 v2.1.0 (Kim et al., 2013). The BAM output of TopHat2 was then pooled across replicates and mapped reads were visualized using IGV v2.3.90 (Robinson et al., 2011; Thorvaldsdottir et al., 2013). RNA-seq data have been deposited in the European Nucleotide Archive^5^ under study accession PRJEB28127.

### Phylogenetic Trees

Phylogenetic trees of *Xoo* ICMP 3125^T^, *Xoo* PXO142, and 15 further *Xoo* strains including *Xoo* AXO1947 as an outgroup were determined (i) based on their TALEs using the “TALE Class Presence” tool of AnnoTALE and (ii) using bcgTree (Ankenbrand and Keller, 2016) based on the protein alignments of its default set of 107 conserved genes.

For the TALE-based phylogenetic tree, distances between *Xoo* strains were determined as the sum of (a) the divergence score of the RVD-based alignment of TALEs in the same AnnoTALE class (Grau et al., 2016), (b) the divergence score of the most similar TALE of the respective other strain if two strains do not comprise TALEs from the same AnnoTALE class, or (c) a divergence score of 6 if no matching TALE exists (chosen to be larger than the cut height of 5 leading to AnnoTALE classes). The matrix of pairwise distances between *Xoo* strains served as input of agglomerative hierarchical clustering using single linkage as implemented in the Jstacs library (Grau et al., 2012) to yield the phylogenetic tree. This procedure is available as part of the “TALE Class Presence” tool since AnnoTALE version 1.3.

The second phylogenetic tree was computed using bcgTree (Ankenbrand and Keller, 2016) based on a set of 107 essential genes as described in Dupont et al. (2012) represented as HMMs. Internally, bcgTree uses multiple other tools, namely hmmsearch (Eddy, 2009) for searching matches to the HMM models, MUSCLE (Edgar, 2004) and Gblocks (Castresana, 2000) for aligning sequences as identifying conserved blocks, and RaxML (Stamatakis, 2014) for computing phylogenetic trees. The bcgTree wrapper was run with default parameters, specifying 200 bootstraps for RaxML. The final phylogenetic tree was then extracted from the “RaxML_bestTree.final” output of bcgTree.

Both trees were visualized comparatively using phylo.io (Robinson et al., 2016).

### Southern Blot

Genomic DNA from *Xoo* was isolated using phenol/chloroform extraction, digested with *Bam*HI and separated on a 0.8% agarose gel at 90 V for 24 h. The genomic TALE sequences were detected with chemiluminescence on Southern blot using a digoxigenin-labeled (Roche Applied Science, Mannheim, Germany) probe derived from 500 bp of the 3′ part of *talC* from *Xoo* BAI3 (Yu et al., 2011) that hybridizes to TALE genes.

### TALE Expression Constructs

TALEs were constructed using the Golden TAL technology kit (Geißler et al., 2011). Individual repeats were subcloned into hexa-repeat modules and subsequently assembled to final TALE expression constructs. N- and C-terminal parts of Hax3 were employed for expression constructs (pSKA2) used in *A. tumefaciens*. Expression constructs for *Xanthomonas* harbored the N-terminal region from TalAG4 of strain PXO83 and the C-terminal region from TalAO3 of strain PXO83. The *Xanthomonas* expression vector (pSKX1) fuses a C-terminal FLAG Epitope to the TALE.

### Western Blot

The *Xoo* strains carrying a plasmid encoding an artificial TAL effector, or empty vector, were grown in liquid PSA medium supplemented with 20 μg ml^-1^ gentamicin at 30°C. Cells of 1 ml of a bacterial suspension at an OD_600_ of 0.2 were harvested and the TAL effector expression was analyzed by sodium dodecyl sulfate polyacrylamide gel electrophoresis (SDS–PAGE) and immunoblotting using an anti–FLAG antibody (Sigma–Aldrich).

### Virulence Assay

The third leaves of 4-week-old *Oryza sativa* ssp. *japonica* cv. Nipponbare plants were clipped with *Xoo* bacterial solution adjusted to an OD_600_ of 0.2 in 10 mM MgCl_2_. 14 dpi leaves were harvested and lesion length was measured.

### RNA Isolation and qRT-PCR

At two dpi, 5 cm inoculated segments were harvested and rice total RNA was isolated using the Qiagen RNeasy kit. cDNA was generated from 2 μg RNA using the Fermentas first-strand cDNA synthesis kit (Thermo Fisher Scientific Inc., Waltham, MA, United States) and real-time PCR was performed using the iCycler (Bio-Rad, München, Germany) as described before (Streubel et al., 2013 and Supplementary Table S6). The amplification efficiency for each primer pair was analyzed using a standard curve plot of a dilution series. cDNA amounts were normalized using actin as a reference gene. The fold change induction was calculated in comparison to leaves treated with 10 mM MgCl_2_ by using the ΔΔCt method (Livak and Schmittgen, 2001). All experiments were repeated three times.

### β-Glucuronidase (GUS) Reporter Constructs and GUS Activity Analysis

β-Glucuronidase assays from plant samples were performed essentially as described before (Boch et al., 2009). Briefly, PCR-amplified fragments of the promoters were cloned into a Golden Gate-compatible pGWB3 (Nakagawa et al., 2007) derivative containing a promoterless *uidA* reporter gene (Supplementary Table S6). To analyze reporter activity, *A. tumefaciens* strains delivering TALE constructs and GUS reporter constructs were resuspended in infiltration medium, resulting in an OD_600_ of 0.8, mixed in equal amounts, and inoculated into *N. benthamiana* leaves. Two dpi, leaf disks were sampled and GUS activities were quantified using 4-methylumbelliferyl-β-D-glucuronide (MUG). Total protein concentrations were determined using Bradford assays. Leaf disks for qualitative staining were harvested in parallel. Histochemical staining was performed using 5-bromo-4-chloro-3-indolyl β-D-glucuronide (X-Gluc). Data were compiled from triplicate samples originating from different plants. All experiments were repeated three times.

## Results

### Complete TALE Repertoires in *Xoo* Strains From India and the Philippines

**FIGURE 1:**
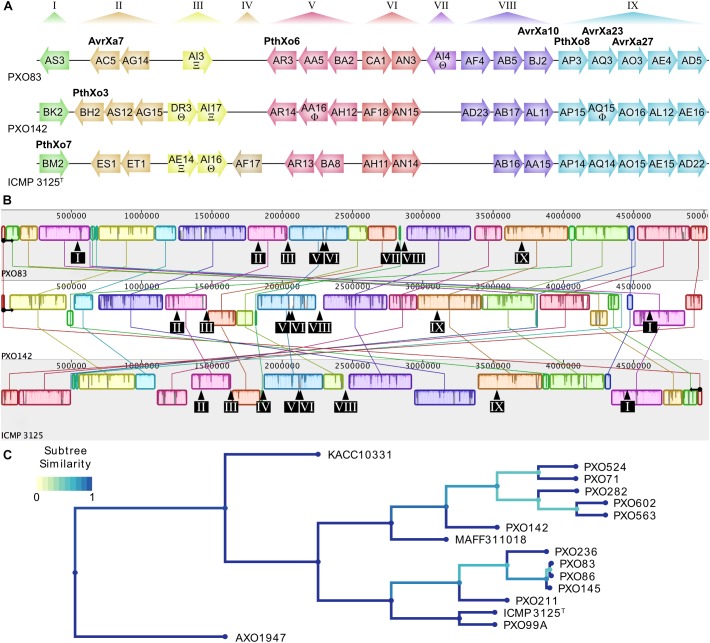
Genomic TALE distribution and genome alignment of *Xoo* strains used in this study. **(A)** TALome overview of previously published *Xoo* strain PXO83 and the newly sequenced strains PXO142 and ICMP 3125^T^. *TALE* genes are represented by arrows indicating their relative orientation in the genome. All TALEs are assigned into classes by AnnoTALE and named accordingly, while the alternative name of prominent members of the classes is indicated in bold. Truncated TALEs (truncTALEs/iTALEs) are labeled with xi (Ξ) for truncTALE-related/iTALE type A or theta (Θ) for truncTALE/iTALE type B. Other TALE pseudogenes without functional N- or C-terminal regions are marked with a phi (Φ). Previously established TALE clusters are specified at the top and cluster affiliation of individual TALE genes is shown by color. **(B)** Progressive MAUVE whole genome alignment of individual *Xoo* strains. Blocks of the same color indicate similar genomic regions. Origin of replication and orientation of *dna*A are indicated by a black circle with an arrow. Due to a large inversion in ICMP 3125^T^, the reverse complement of the genome is used for easier alignment. The locations of TALE clusters are shown by black arrows and boxes with cluster numbers. **(C)** Phylogenetic tree of all fully sequenced Asian *Xoo* strains. All fully sequenced Asian *Xoo* strains were used to create a phylogenetic tree based on TALE RVD sequences. The African *Xoo* strain AXO1947 was used as an outgroup. The color scale of “Subtree Similarity” indicates the Jaccard index between sets of subtree leaves.

To compare the TALomes of *Xoo* strains from different agricultural regions, we sequenced the strains PXO142 and ICMP 3125^T^, originating from the Philippines and India, respectively. The complete genome sequences of both strains were obtained using PacBio sequencing, which produces long reads that are suitable to correctly assemble *TALE* genes in their genomic context. The genomes of *Xoo* PXO142 and ICMP 3125^T^ were assembled into single contigs of 4.99 Mbp length, each (Table 1 and Supplementary Figures S1, S2). These genomes were compared to the other available Asian *Xoo* genomes and phylogenetic trees were created based on conserved genes and TALE RVD sequences (Figure 1C and Supplementary Figure S3). Using the AnnoTALE prediction pipeline, *TALE* genes were identified in these genomes and assigned to individual TALE classes. At present, the total number of 278 Asian *Xoo* TALEs from the fully sequenced strains fall into 38 different classes^6^. PXO142 and ICMP 3125^T^ contain 19 and 17 *TALE* genes, respectively, which were assigned to 23 different TALE classes (Figure 1A and Supplementary Table S3). Eleven of these classes are shared by both strains whereas the remaining 12 are only present in one of the strains. Six identified TALE classes have a prominent member that has previously been described in the context of resistance reactions – AvrXa23 (TalAQ) and AvrXa27 (TalAO) – or as an important virulence factor – PthXo3 (TalBH), PthXo6 (TalAR), PthXo7 (TalBM), and PthXo8 (TalAP) (Figure 1). The location of *TALE* genes in both strains is confined to the previously described TALE clusters T-I to T-IX (Figure 1). The MAUVE alignment of the sequenced genomes (Figure 1B) revealed a high level of genomic rearrangements, which is typical for plant pathogenic bacteria (Darling et al., 2004). TALE clusters T-III, T-IV, T-VII, and T-VIII are in the direct vicinity of rearrangements, whereas the other clusters are harbored in more stable genomic regions (Figure 1B).

### TALome Comparison – Clustering and TALE Abundance

**FIGURE 2:**
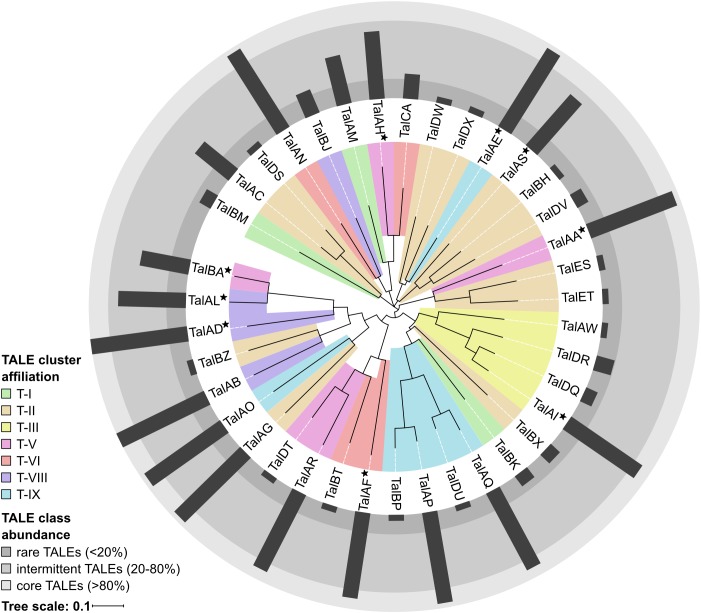
TALE class abundance and cluster affiliation. A phylogenetic tree of all 38 Asian *Xoo* TALE classes was created by aligning the most likely common binding sequences of each TALE class with Clustal Omega and visualization was done using iTol. TALE cluster affiliations of each TALE class are represented by the colored background of the respective branches. If the TALE class is present in different TALE clusters the most abundant location is shown and the class is marked with a star. The abundance of each TALE class in Asian *Xoo* strains is shown as a radial bar diagram and the three assigned abundance categories are depicted as different shades of gray.

In order to evaluate the TALome diversity in all 16 sequenced Asian *Xoo* strains, the TALE classes were assigned to three abundance categories depending on how frequently they occur (Figure 2). Core TALE classes were defined as being present in more than 80% of strains, intermittent TALE classes in 20–80% of strains, and rare TALE classes in less than 20% of strains (Figure 2). Notably, *TALE* genes with truncated N- or C-terminal regions do not bind to DNA and do not activate gene expression, respectively. Some of these TALEs block resistance reactions and have been termed truncTALEs or iTALEs and were classified, accordingly. Other truncated TALEs were categorized as pseudogenes. PXO142 contains nine core TALEs, three intermittent TALEs, three rare TALEs, two pseudogenes, one truncTALE/iTALE type B and one truncTALE-related/iTALE type A. ICMP 3125^T^ carries ten core TALEs, two intermittent TALEs, three rare TALEs, one truncTALE/iTALE type B and one truncTALE-related/iTALE type A. Interestingly, TalES1 and TalET1 (both in cluster T-II) of ICMP 3125^T^ are unique and have no other class members, so far.

Our analysis revealed that the TALE clusters T-I to T-III contain the majority of rare and intermittent TALE classes and are highly diverse in their composition. Therefore, these clusters have the highest potential for new TALEs to be discovered in the future. On the contrary, T-IV to T-IX are highly conserved in their TALE class content and contain a high amount of core TALEs. The consistency of these clusters suggests that the TALEs in these clusters play an important role in *Xoo* infection.

A phylogenetic tree of all 38 different TALE classes from 16 *Xoo* strains was created by aligning the most likely common binding sites for each TALE class with Clustal Omega and visualization was done using iTol (Figure 2) (Sievers et al., 2011; Letunic and Bork, 2016). Apparently, some rare TALE classes are related to core TALE classes present in a different strain at the same genomic locus. Examples are the TALE classes TalAQ and TalDU, TalAC and TalDS, as well as TalAR and TalDT (Figure 2), which are mutually exclusively present in the same genomic location in different strains (Supplementary Figure S4). Evidently, the deletion of one to three repeats triggered a separate classification by the AnnoTALE software, because the insertion or deletion of repeats typically has a significant impact on TALE binding. As TalAQ (AvrXa23) and TalAC (AvrXa7) trigger a resistance reaction in rice cultivars carrying the *Xa23* and *Xa7* resistance loci, respectively (Yang and White, 2004; Wang et al., 2015), the deletion of repeats could circumvent recognition by the plant. Alternatively, these changes could be adaptations to indel mutations in target promoter sequences in different rice cultivars (Richter et al., 2014; Erkes et al., 2017). Using the daTALbase tool (Pérez-Quintero et al., 2018) we searched for variations in the TALE boxes of TalAQ, TalAC, and TalAR in the promoters of their target genes, *OsFNS, OsSWEET14*, and *OsTFX1*, respectively, but no variations were found.

All truncTALE/iTALEs are closely related in their RVD sequences, suggesting a common origin. TALE class TalDR is exclusively a truncTALE/iTALE type B and TALE class TalAW is exclusively a truncTALE-related/iTALE type A. TALE classes TalDQ and TalAI have *TALE* genes of either truncTALE/iTALE type.

### Potential Targets – Combining *in silico* and *in vivo* Methods

**FIGURE 3:**
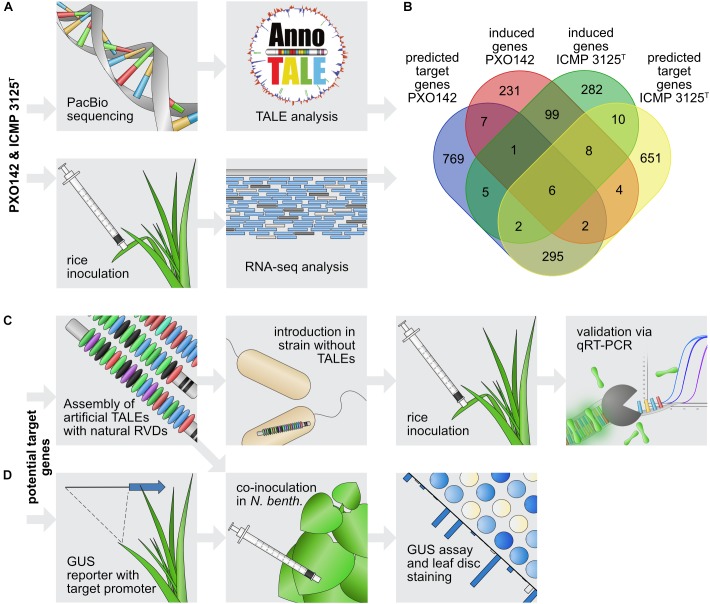
Workflow of TALE target gene identification. **(A)** Identification of candidate target genes. The top panel describes the steps for *in silico* predictions and the bottom panel shows the *in vivo* transcriptome analysis. **(B)** Venn diagram of intersections between predicted target genes and genes induced upon *Xoo* infection. **(C,D)** Strategy to evaluate individual candidate target genes. **(C)** Steps to identify the influence of single TALEs on target gene expression. **(D)** Steps to assess interactions between individual TALEs and their respective target promoters.

In order to identify plant target genes of different TALEs, we combined *in silico* target predictions with *in vivo* gene expression data. The top 100 target sequences for each TALE from *Xoo* PXO142 and ICMP 3125^T^ were predicted using TALgetter (Grau et al., 2013). The promoterome of the cultivar Nipponbare was restricted to 300 bp upstream and 200 bp downstream of transcriptional start sites, which is the most promising region for TALE binding sites (Grau et al., 2013). Overall 2,430 unique potential target genes were predicted for both strains combined, of which 430 were identified for both strains (Figure 3A).

To identify real TALE-induced genes among the *in silico* predicted target candidates, the expression of rice genes was determined following infection with *Xoo*. The rice cultivar Nipponbare was inoculated with PXO142, ICMP 3125^T^, or a mock control, and harvested after 24 h for subsequent RNA-seq analysis. This relatively early harvest time was chosen to favor primary TALE targets and minimize secondary effects at the risk of missing later changes. Comparing gene expression in infected and uninfected tissue enables the identification of *Xoo*-mediated gene induction. Differences in the TALE repertoire of these *Xoo* strains should enable the comparison of TALE class presence and absence. The RNA-seq analysis yielded 358 and 413 induced rice genes (≥1.5-fold) during infection with PXO142 and ICMP 3125^T^, respectively. One hundred and fourteen of these rice genes were induced by both strains. Thirty-six promising target gene candidates for 15 TALE classes were identified by combining *in silico* prediction and RNA-seq analysis (Supplementary Table S2 and Figure 3B).

### Assigning Targets for Individual TALEs

In order to assign induced plant genes to individual TALEs, *Xoo* strains expressing only one *TALE* gene were created and their impact on target gene expression was monitored (Figure 3C). To this end, the American *Xoo* strain Roth X1-8, which has no natural TALEs (Supplementary Figure S5), was transformed with expression plasmids carrying individual *TALE* genes. *TALE* expression constructs were created by using the Golden TAL Technology cloning Kit (Geißler et al., 2011). To facilitate the exact replication of the natural RVD composition in the artificial TALEs, we expanded the original cloning kit with additional RVD modules and assembly vectors (Supplementary Figure S6). Furthermore, to match the artificial TALEs as closely as possible to their natural counterparts, we used *Xoo*-specific N- and C-terminal regions (Supplementary Figures S7, S8). Twelve different Roth X1-8 derivatives were created carrying single TALEs ranging from 11.5 to 28.5 repeats. The production of the individual TALEs was verified via Western Blot (Supplementary Figure S9). The impact of single TALEs on the virulence of the TALE-less *Xoo* strain Roth X1-8 was tested by clipping inoculation of Nipponbare leaves with bacterial solution (Supplementary Figure S10). TalBH2 supported bacterial growth, which was expected, because it induces expression of the well-known *OsSWEET14* virulence target. The other TALEs analyzed showed no clear contribution to virulence under the tested conditions.

To analyze expression of target genes, rice leaves were inoculated with *Xoo* strains and samples were taken for RNA isolation and qRT-PCR analysis. The rice variety Nipponbare was inoculated with Roth X1-8, Roth X1-8 carrying single *TALE* expression constructs, the wild type strains PXO142, ICMP 3125^T^, PXO83, and a mock control. *Xoo* strain PXO83 was previously sequenced and the analyzed TALome is available (Grau et al., 2016). As PXO83 has members of the same TALE classes as PXO142 and ICMP 3125^T^, we expected a similar induction in common predicted target genes. Samples were collected after 2 days to facilitate robust assessment of gene induction via qRT-PCR. For five TALE classes (TalAE, TalAF, TalAG, TalAN, TalET) no target candidates were identified in the combinatorial target prediction and RNA seq analysis. Furthermore, for TalAL, the only identified candidate (a retrotransposon) was not very promising. In these cases we chose other candidates from the target prediction list for qRT-PCR tests.

**FIGURE 4:**
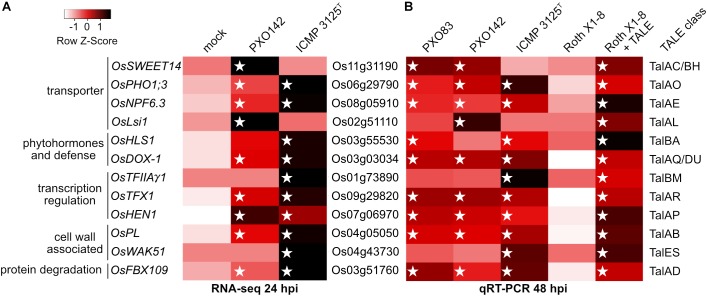
Induction of target rice gene expression upon infection with *Xoo* wild type strains and *Xoo* Roth X1-8 expressing single artificial TALEs. Unclustered heat maps were created using Heatmapper and rice gene expression is displayed via row Z-scores. Individual rice genes are labeled with their locus identifier (middle), gene name and putative function (left). The right column indicates TALE classes with target boxes in the respective rice promoters. White stars mark the presence of a TALE class member in the different *Xoo* strains. **(A)** TALE-mediated gene induction analyzed via RNAseq with RNA sampled 24 h after inoculation of Nipponbare. Z-scores were assigned to arithmetic means of resulting RNAseq reads of the three replicates and displayed in shades of red. **(B)** Gene induction level analyzed via qRT-PCR with RNA sampled 48 h after inoculation of Nipponbare. The wild type *Xoo* strain Roth X1-8 does not contain any TALEs. Z-scores of relative RNA abundance were assigned to arithmetic means of two biological replicates using log_10_ fold changes of gene expression in samples compared to mock treatment and displayed in shades of red. Actin was used as a reference gene.

We were able to match twelve specific target genes to TALE classes and confirmed these as TALE-dependently induced genes (Figure 4 and Supplementary Table S4). All 12 target genes were induced significantly in a comparison between strain Roth X1-8 with or without the corresponding TALE with *p*-values below 0.05.

Three TALE targets were known previously: *OsSWEET14, OsTFIIAγ1*, and *OsTFX1* are induced by TALE classes TalBH, TalBM and TalAR, respectively. Five TALE targets were only hypothesized to be TALE targets before and are now experimentally confirmed: *OsLsi1, OsHEN1, OsPHO1;3, OsNPF6.3* and *OsFNS* are addressed by TALE classes TalAL, TalAP, TalAO, TalAE and TalAQ, respectively. Finally, four identified TALE targets have not been described before: *OsPL, OsWAK51, OsHLS1* and *OsFBX109* are manipulated by TALE class TalAB, TalES, TalBA, and TalAD. The target genes *OsLsi1* of TalAL and *OsNPF6.3* of TalAE were ambiguous in our RNAseq experiment at 24 hours post inoculation (hpi), but clearly induced in our qRT-PCR experiment at 48 hpi. This observation suggests that some TALEs might have a delayed effect within the host cell that is more pronounced at 48 hpi than at 24 hpi.

**FIGURE 5:**
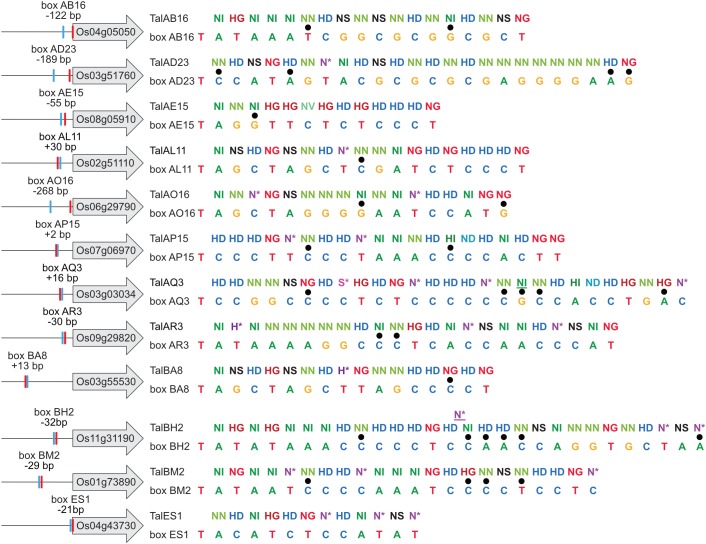
TALE boxes in rice target gene promoters. TALE target genes in rice cultivar Nipponbare with 1,000 bp upstream of their ATGs are shown schematically on the left and labeled with their locus identifiers. Locations of TALE boxes are indicated by blue boxes and positions relative to the annotated transcription start site (red box) are noted. RVD sequences of natural TALEs analyzed in this study are lined up with the sequences of best fitting target boxes. Mismatches are distinguished by black dots. TalAQ3 and TalBH2 have a repeat with 42 and 39 amino acids, respectively. Such repeats of aberrant length can either insert into the repeat array or loop out to accommodate 1 bp shorter target sequences. Aberrant repeats are underlined.

The predicted TALE boxes in the promoters of the target genes match well to the RVD-DNA base specificities (Figure 5). They are located on average 240 bp upstream of the start codon with outliers very close to the start codon (TalES1 box 21 bp upstream of OsWAK51 CDS) and quite far from it (TalBA8 box 635 bp upstream of OsHLS1 CDS) (Figure 5). Most TALE boxes are also very close to the annotated natural transcription start site with the average TALE box located 57 bp upstream (Figure 5). This distance is well suitable because TALEs typically determine the onset of transcription in a distance of 40–60 bp after their binding site. In contrast, the TALE boxes of TalAB16, TalAD23, and TalAO16 are located 122, 189, and 268 bp upstream of the natural transcription start sites, respectively (Figure 5). Accordingly, the apparent transcription start site of *OsPHO1;3* in plants infected with *Xoo* strains carrying TALE TalAO appears to be shifted in comparison to the uninfected tissue in our RNA-seq data (Supplementary Figure S11). This supports the observation that TALEs can control the transcriptional start site of induced target genes. The TALE boxes had a mean mismatch rate of 11% with TalES1 fitting the promoter perfectly and TalBH2 tolerating six mismatches out of 29 bases.

The expression patterns of all twelve genes during infection coincide with the presence/absence of their corresponding TALE class in a given *Xoo* strain. This suggests that the TALE class affiliation is a reliable indicator of TALE function. The specific functions of 67% of all known TALEs of Asian *Xoo* strains can now be predicted because they belong to a TALE class containing a member with a known target gene.

### Direct Recognition of Target Promoters

We aimed to determine whether the TALEs directly or indirectly induced the identified target rice genes. Direct induction would involve binding of the TALE to the target promoter whereas indirect induction could be mediated via secondary effects or secondary regulatory rice genes. To distinguish this, transient GUS reporter assays were performed in the heterologous plant *Nicotiana benthamiana* using individual TALEs and subcloned rice promoters. A GUS activity would imply a direct recognition of the target promoter by the TALE because secondary targets would not be present in *N. benthamiana* (Figure 3D).

**FIGURE 6:**
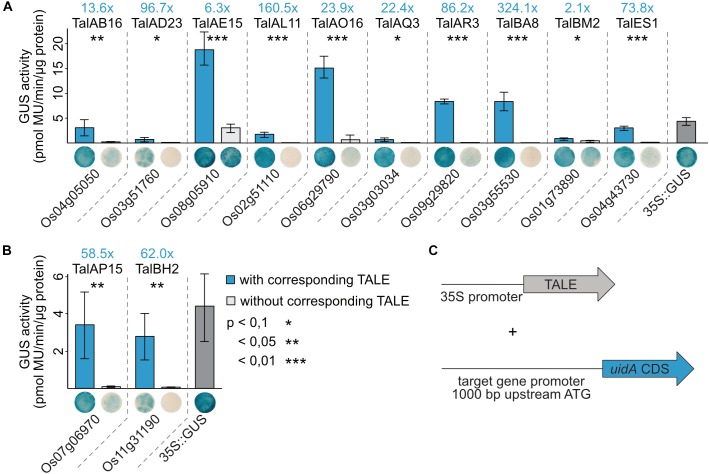
TALEs directly induce expression of target rice promoters in reporter studies in *N. benthamiana*. **(A,B)** 1,000 bp upstream of the ATGs of the TALE target genes were amplified from rice cultivar Nipponbare DNA and cloned in front of a promoterless *uidA* reporter gene. Artificial TALEs were assembled with RVD sequences shown in Figure 5 and Hax3 N- and C-terminal regions under control of a 35S promoter. *A. tumefaciens* strains delivering the reporter constructs and strains delivering the TALE expression constructs were co-inoculated into *N. benthamiana* leaves and β-glucuronidase measurements were performed 2 dpi. Quantitative GUS activity measurements were performed three times with samples obtained as described above. Error bars represent standard deviation between triplicates. The statistical significance between samples with and without corresponding TALEs is indicated by *p*-values resulting from an unpaired *t*-test and fold changes are marked in blue. The TALE Hax3 is used as a negative control in samples labeled without corresponding TALE. Histochemical GUS staining of leaf disks and quantitative GUS activity measurements were done in parallel from the same plants. Leaf disks were stained in GUS staining solution and destained in 96% ethanol. One representative leaf disk is shown. **(C)** Schematic overview of experimental setup.

1,000 bp upstream of the start codon of the twelve identified rice target genes were amplified from rice cultivar Nipponbare DNA and cloned in front of a promoterless *uidA* coding sequence. Artificial TALEs were constructed with the same RVD composition as the natural TALEs (Figure 5) and cloned into *Agrobacterium* binary vectors. The GUS reporter constructs and the TALE expression constructs were introduced into *Agrobacterium tumefaciens* and the respective strains were co-inoculated into *N. benthamiana*. After 2 days, samples were harvested for quantitative and qualitative GUS assays (Figure 6).

Most reporter constructs show very little GUS activity without a TALE indicating that the basal expression rates of these rice promoters in *N. benthamiana* are low. Only *OsNPF6.3* (Os08g05910) shows a relatively pronounced GUS activity without TalAE15. The GUS activity in samples with the corresponding TALEs compared to controls was significantly higher for all 12 tested target genes. The GUS activity of the different reporter constructs varied with *OsNPF6.3* (Os08g05910), *OsPHO1;3, OsTFX1*, and *OsHLS1* reporter constructs showing the highest absolute GUS activity when paired with their matching TALEs (Figure 6). This corresponds to induction rates of 10- to 100-fold for most combinations. The only exceptions are *OsNPF6.3* and *OsTFIIAγ1* with less than 10-fold increases in GUS activity and *OsLsi1* and *OsHLS1* with an increase of more than 100-fold (Figure 6). These results indicate that the examined TALEs are able to directly recognize the identified promoters *in vivo* and trigger gene expression.

### Convergent TALEs

**FIGURE 7:**
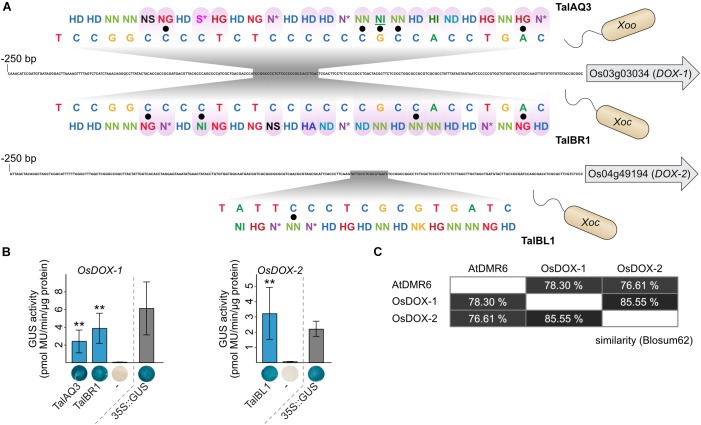
Functional convergence of TALEs from *Xanthomonas oryzae*. **(A)** Functional convergence of TALEs on rice *OsDOX-1* and *OsDOX-2*. 250 bp upstream of the start codon of target genes are displayed and TALE boxes are highlighted in gray. TALE boxes are matched to the RVD sequences of the corresponding TALEs. TalAQ has a repeat of aberrant length (42 amino acids) that is underlined. Mismatches are indicated by black dots. Differences in RVD sequences between TALEs with the same TALE box are distinguished by a purple tint. **(B)** 1,000 bp upstream of the ATGs of the TALE target genes from rice cultivar Nipponbare DNA were cloned in front of a promoterless *uidA* reporter gene. *A. tumefaciens* strains delivering the reporter constructs and strains delivering the TALE expression constructs were co-inoculated into *N. benthamiana* leaves and β-glucuronidase measurements were performed 2 days after infiltration. Error bars represent standard deviation between triplicates. The experiment was done three times with comparable results. The statistical significance between samples with and without corresponding TALEs is indicated by *p*-values resulting from an unpaired *t*-test (^∗^*p* < 0.1; ^∗∗^*p* < 0.05; ^∗∗∗^*p* < 0.01). The TALE Hax3 was used as a negative control (-). GUS staining of leaf disks and quantitative GUS activity measurements were done in parallel from the same plants. One representative leaf disk is shown. **(C)** Amino acid similarities are based on Blosum62.

Key virulence targets that are important for a successful infection are often addressed by multiple virulence factors across pathogen species and host plants. One of the best-studied examples of such a convergent evolution for TALE targets are *SWEET* genes. *SWEET* genes in rice, cassava, sweet orange, and cotton are induced by different TALEs from different *Xanthomonas* strains (Yang and White, 2004; Cohn et al., 2014; Zhou et al., 2015; Cox et al., 2017). Here, we propose two new cases in which TALEs from different origin converge on candidate virulence targets. The first target family consists of the 2-oxoglutarate dioxygenase (DOX) genes *OsFNS* (Os03g03034) and Os04g49194. These genes share 69.6% identity and 85.6% similarity (Figure 7C and Supplementary Figure S12). They are closely related homologs that are predicted to convert flavanones to flavones, but the experimental evidence is contradictory (Kim et al., 2008; Lam et al., 2014; Falcone Ferreyra et al., 2015; Zhang et al., 2017). Our target analysis revealed that Os03g03034 is induced by *Xoo* TalAQ (Figure 4, 6). In addition, induction of both genes by *Xoc* has been proposed by TALE target predictions and transcriptomic data (Cernadas et al., 2014), but experimental validation for a direct induction is lacking. The binding specificities of the *Xoc* TALE classes TalBR and TalBL match well to the promoter regions, respectively (Figure 7A). We re-constructed one *Xoc* TALE of each class and measured recognition of the respective target promoters in GUS reporter assays (Figure 7B). These experiments show that three distinct TALE classes of different *X. oryzae* pathovars are targeting these rice genes. *Xoo* strains address Os03g03034 with TALE class TalAQ, whereas *Xoc* strains target Os03g03034 and Os04g49194 with TALE classes TalBR and TalBL, respectively. Interestingly, TalAQ and TalBR have the exact same binding sequence in the Os03g03034 promoter even though 16 out of their 27 RVDs differ (Figure 7A). The functional relationship between both targets has so far been overlooked. To acknowledge this connection, we propose to rename Os03g03034 to *OsDOX-1* and Os04g49194 to *OsDOX-2*.

The second new target of convergent TALE classes is the gene *OsLsi1* (Os02g51110) which encodes a putative silicon transporter (Ma et al., 2006), and which has been identified by us as a *Xoo* TalAL target (Figure 4, 6). This gene was also previously hypothesized to be a target of the TALE class TalAV (Erkes et al., 2017). We re-constructed *Xoc* TalAV1 and tested the direct recognition of the target promoter in a GUS-assay (Supplementary Figure S13). This experiment shows that the promoter of *OsLsi1* is directly recognized by TALE classes TalAL from *Xoo* and TalAV from *Xoc*. The two TALE classes bind different positions in the promoter, suggesting a co-evolution. PXO142 might even be adapted to different versions of this promoter, as it has two members of TALE class TalAL with minor differences in their RVDs (Supplementary Table S5). According to the daTALbase, there is a known variant of the *OsLsi1* promoter with the altered TALE box TAG[C/T]TAGCTCGATCTCCCT, but the SNP does not correspond to differences between the TALE class TalAL members.

These two examples demonstrate that *Xoo* and *Xoc* have more common infection strategies than previously believed.

## Discussion

**FIGURE 8:**
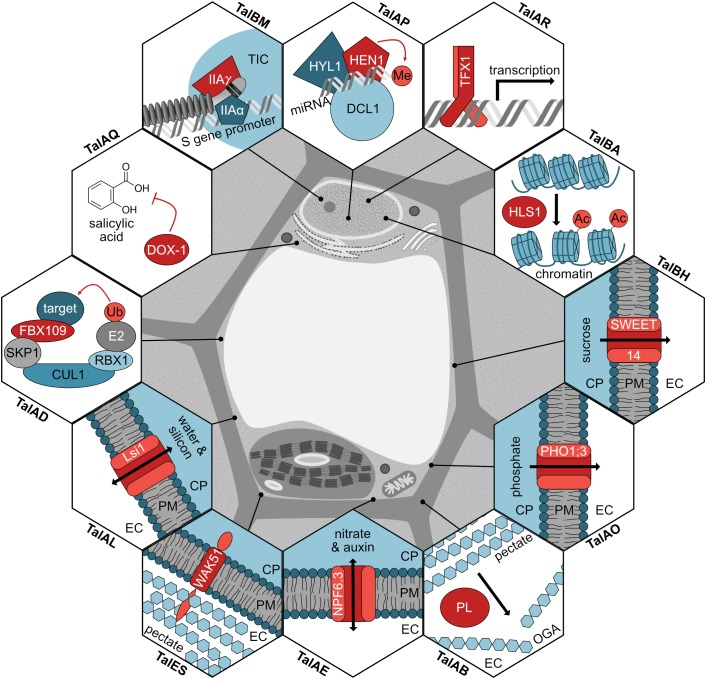
Modes of action for TALEs in rice. The function for each TALE target (red) is shown in a hexagon, the subcellular location of the proteins is shown, and the corresponding TALE classes inducing the genes are noted. If the TALE target modifies a substrate, the modification is displayed in light red. TIC, transcription initiation complex; miRNA, microRNA; Me, methyl group; Ac, acetyl group; CP, cytoplasm; PM, plasma membrane; EC, extracellular space; PL, pectate lyase; OGA, oligogalacturonides; Ub, ubiquitin.

In this work, we significantly broaden the knowledge of TALE-mediated plant manipulation by *Xoo*. A detailed picture of common *Xanthomonas* virulence strategies is emerging (Figure 8) and new insight into functional convergence between different pathovars is gained.

### Asian *Xoo* Have a Common Core Set of TALEs

The strains ICMP 3125^T^ and PXO142, which were sequenced in this study, expand the variety of fully sequenced Asian *Xoo* to 16 strains originating from India, the Philippines, Japan, Korea, and Taiwan (Ochiai et al., 2005; Salzberg et al., 2008; Streubel et al., 2013; Booher et al., 2015; Quibod et al., 2016; Char et al., 2018; NZ_CP011532.1). The isolations span a time from 1965 to 2006 for ICMP 3125^T^ and PXO602, respectively (Ochiai et al., 2005; Salzberg et al., 2008; Streubel et al., 2013; Booher et al., 2015; Quibod et al., 2016; Char et al., 2018; NZ_CP011532.1). With these resources, a spatiotemporal view of Asian *Xoo* strains can now be established. In order to get a comprehensive picture of differences and similarities between strains, a variety of tools was applied. At first, we characterized the TALomes of all strains with AnnoTALE by assigning TALEs into 38 TALE classes. The TALE classes represent groups of TALEs with similar RVD sequences and subsequently common binding sequences and target genes (Grau et al., 2016). Next, we assessed the frequency of each TALE class in those 16 strains. We identified 12 core TALE classes present in over 80% of strains, 18 rare TALE classes found in fewer than 20% of strains and eight intermittent TALE classes occurring in 20–80% of strains. Core TALE classes have a high prevalence and are therefore likely to be generally important for the infection, as they are conserved over a long time and different geographical regions. At the same time, rare TALE classes might be involved in adaptations to different climatic regions or regional rice cultivars. One example is the rare TALE class TalBM targeting *OsTFIIAγ1*, which only promotes virulence during infection of rice cultivars with a *xa5* genomic background (Sugio et al., 2007). Additionally, we found some rare TALE classes that are closely related to core TALE classes, which might circumvent resistances by altering existing *TALE* genes to evade detection or to overcome promoter mutations.

### Unraveling Core TALE Targets in Rice

In order to investigate the targets of the TALE classes present in the strains ICMP 3125^T^ and PXO142, we combined *in silico* target predictions with *in vivo* transcriptomic data. Our analyses revealed 36 potential target genes that were TALE-dependently induced and contained a predicted TALE box in their promoter region. Of these, 12 could be verified and attributed to individual TALEs. Among these target genes were the three previously published targets *OsSWEET14, OsTFIIAγ1*, and *OsTFX1* for TALE class TalBH, TalBM, and TalAR, respectively (Sugio et al., 2007; Streubel et al., 2013). Additionally, we provide experimental data to support the relationship between five more TALE classes with their previously hypothetical target genes *OsLsi1* (TalAL), *OsHEN1* (TalAP), *OsPHO1;3* (TalAO), *OsNPF6.3* (TalAE), and *OsDOX-1* (TalAQ) (Grau et al., 2013; Cernadas et al., 2014). Finally, we introduce four new TALE target genes, that have not been described before: *OsPL* (TalAB), *OsWAK51* (TalES), *OsHLS1* (TalBA), and *OsFBX109* (TalAD). Because different members of the TALE classes were able to induce the same target genes, the consistency between TALE classes and common target genes could be shown. Taken together, this broadens our knowledge of *Xoo* TALE target genes significantly with now 60% of all functional Asian *Xoo TALE* genes having a known target (Yang and White, 2004; Yang et al., 2006; Chen et al., 2010; Streubel et al., 2017; Tran et al., 2018). Especially the targets of core TALE classes are brought to light, as we expand the number of experimentally confirmed targets from one (*OsTFX1*) to seven (*OsTFX1, OsPL, OsFBX109, OsNPF6.3, OsPHO1;3, OsHEN1, OsDOX-1*) out of eleven. Addressing targets of core TALEs might be the most sustainable way to provide resistance against a variety of Asian *Xoo* strains.

### *Xoo* Infection Induces Expression of Specific Pathogenicity Hubs

It can be expected that several of the confirmed and postulated target genes have relevance in *Xoo* infection of rice. In fact, some of them can be grouped into known pathogenicity hubs often manipulated by *Xanthomonas*. In the following, possible virulence implications of selected TALE targets are discussed.

#### Coerced Nutrient Suppliers

The first group of target genes contains transporters that could provide the pathogen with nutrients. Among these is the well-described sugar exporter OsSWEET14 (Os11g31190; target of TalBH), which is known to be a widespread virulence target for TALEs (Yang and White, 2004; Yang et al., 2006; Antony et al., 2010; Römer et al., 2010; Yu et al., 2011; Streubel et al., 2013; Cohn et al., 2014; Zhou et al., 2015; Cox et al., 2017). The export of sugar is thought to lead to nutrient accumulation in the apoplast and xylem to promote colonization (Streubel et al., 2013). The phosphate transporter OsPHO1;3 (Os06g29790; target of TalAO) has a well-described homolog in *Arabidopsis thaliana* (AtPHO1) known to be responsible for xylem loading of phosphate (Secco et al., 2010, 2012; Młodzińska and Zboińska, 2016). It is therefore feasible that *Xoo* induces OsPHO1;3 to ensure its phosphate supply during growth in the xylem. Phosphate availability is directly involved in biofilm formation of phytopathogens and uptake of inorganic phosphate was shown to be essential for virulence of *X. citri* pv. *citri* in citrus and *X. axonopodis* pv. *glycines* in soybean (Moreira et al., 2015; Chatnaparat et al., 2016). The transporter OsNPF6.3 (Os08g05910; target of TalAE) is closely related to AtNPF6.3 of *A. thaliana*, which bidirectionally transports nitrate and auxin and promotes stomata opening (Guo et al., 2002, 2003; Krouk et al., 2010; Léran et al., 2014). Accordingly, induction of OsNPF6.3 could lead to nitrate transfer toward the bacteria as well as tampering with the distribution of the phytohormone auxin within the plant. Nitrate assimilation was shown to be important for virulence of the phytopathogen *Ralstonia solanacearum* in the xylem (Dalsing et al., 2015). Auxin has been reported to increase the susceptibility to *Xoo* and *Xoc* in rice by loosening the cell wall through expansin induction (Ding et al., 2008; Fu and Wang, 2011). We previously hypothesized OsPHO1;3 and OsNPF6.3 to be TALE targets because of predictions and expression data of infected tissue (Grau et al., 2013).

The silicon transporter OsLsi1 (Os02g51110; target of TalAL), also known as OsNIP2;1, is a well-studied passive transporter responsible for silicon transport through the casparian bands in rice roots (Ma et al., 2006, 2011). Rice is a silicon accumulator and silicon nutrition is beneficial to rice yield and prevents lodging (Savant et al., 1996; Currie and Perry, 2007). In plant–pathogen interactions, silicon has been described to elicit broad-spectrum disease resistance in different plants, but the experiments often used silicon starvation as a reference point and the mechanism behind the observed effect is not known (Van Bockhaven et al., 2013). Rice plants grown without silicon were more susceptible to *Xoo*, including impaired defense gene expression and lower lignin and phenolics concentrations (Song et al., 2016). The *OsLsi1* homolog *OsLsi6*, which is expressed in xylem parenchyma, was shown to be important for xylem unloading and deposition of silicon (Yamaji et al., 2008). Overexpressing *OsLsi1* at the infection site might relocate silicon and reduce the associated defense reactions. As the details of the relationship between silicon and defense are yet to be uncovered, the consequences of *Xoo*-mediated induction of a silicon transporter are still speculative. Interestingly, we were able to show functional convergence between *Xoo* and *Xoc* in addressing *OsLsi1*, which is a previously unknown TALE target.

Taken together, *Xoo* TALEs redirect the flow of several compounds in and out of plant cells in the infected tissue by transcriptional upregulation of host genes encoding transport proteins.

#### Hormonal Imbalances Provide Easy Prey

The second group of TALE target genes is involved with phytohormone activity and defense regulation, which are attractive targets for pathogens. The N-acetyltransferase OsHLS1 (Os03g55530; target of TalBA) is a homolog of the histone acetyltransferase AtHLS1, which is reported to regulate responses to pathogens and abscisic acid (ABA) by acetylating the chromatin of target loci (Liao, 2016). Inducing a histone acetyltransferase, that loosens the chromatin and boosts transcription, might be generally beneficial to TALE-mediated gene induction (Görisch et al., 2005). AtHLS1 was described to be involved in ABA-mediated priming of plant defenses in necrotrophic pathogens (Liao, 2016). Overexpression of *AtHLS1* lead to hypersensitivity to ABA (Liao, 2016), and amplifying ABA signaling could be beneficial for biotrophic pathogens, as ABA promotes susceptibility to *Xoo* by suppressing SA-mediated defenses in rice (Xu et al., 2013).

The 2-oxoglutarate dioxygenase OsDOX-1 (Os03g03034; target of TalAQ) is closely related to the Arabidopsis gene *AtDMR6* that negatively affects expression of defense genes (van Damme et al., 2008; Kawai et al., 2014; Falcone Ferreyra et al., 2015). Mutation of *AtDMR6* triggers broad-spectrum disease resistance and accumulation of SA (Zeilmaker et al., 2015). Similarly, the deletion of the tomato homolog *Sldmr6-1* renders plants resistant to *X. campestris* pv. *vesicatoria* (Thomazella et al., 2016). As mutation of *AtDMR6* lead to SA accumulation, it was first believed to be an SA-3-hydroxylase (Kawai et al., 2014; Zeilmaker et al., 2015), but AtDMR6 was unable to use SA as a substrate in an enzyme activity assay (Falcone Ferreyra et al., 2015). Instead, flavanones were converted to flavones, suggesting that *AtDMR6* encodes a genuine flavone synthase I (Falcone Ferreyra et al., 2015). In contrast, Zhang et al. (2017) recently showed that the affinity of AtDMR6 to SA as a substrate was significantly higher than to flavanones and referred to AtDMR6 as an SA-5-hydroxylase. These conflicting data make the true enzymatic function of AtDMR6 at present unclear. The corresponding rice enzyme OsDOX-1 was previously described to use the flavanone naringenin as a substrate *in vitro* (Kim et al., 2008). But Lam et al. (2014) reported no measurable flavone synthase I function *in vivo* when *OsDOX-1* was expressed in Arabidopsis. The functional link between the *dmr6* mutation which triggers SA accumulation and the targeting of its rice homologs by *Xoo* and *Xoc* TALEs is compelling. It emphasizes that *Xanthomonas* virulence and plant resistance fundamentally converge at the same plant component suggesting a new hub in the plant–pathogen network.

#### Manipulating the Very Core of the Plant Cell

The third group of TALE targets is comprised of genes taking part in transcriptional regulation, which is a good access point for far-reaching manipulations. The transcription initiation factor IIA γ-subunit (OsTFIIAγ1; Os01g73890; target of TalBM) is a well-known TALE target (Sugio et al., 2007). It was recently shown that the C-terminal domain of TALEs interacts with the TFIIA α+γ subcomplex possibly to replace the TFIIA β subunit in the TFIIA ternary complex and hijack the host transcription machinery (Ma et al., 2018). Induction of *OsTFIIAγ1* is only contributing to virulence of *Xoo* in rice varieties containing a homozygous *xa5* mutation in *OsTFIIAγ5*, which reduces binding of TALEs to OsTFIIAγ5 and interferes with TALE function (Sugio et al., 2007; Yuan et al., 2016; Huang et al., 2017). The bZIP transcription factor OsTFX1 (Os09g29820; target of TalAR) was also shown to be an important susceptibility target since induction of this gene contributes to virulence of *Xoo* in rice (Sugio et al., 2007). The underlying mechanisms are still unclear. The methyltransferase OsHEN1 (Os07g06970) is predicted to methylate the 3′ end of small RNA duplexes, inhibiting their degradation (Achkar et al., 2016). Both micro RNAs and small interfering RNAs are specialized in post-transcriptional regulation by mRNA cleavage or translation repression (Zhang et al., 2006). Especially micro RNAs are hypothesized to need HEN1-dependent methylation to be integrated into RNA-induced silencing complexes (Baranauskė et al., 2015). Plant micro RNAs regulate a plethora of different plant processes making the effects on disease development difficult to identify (Zhang et al., 2006; Samad et al., 2017).

#### Tearing Down the Walls

The fourth group of TALE targets is comprised of genes that can alter the cell wall or perceive alterations, which may allow easier access to the host cell if modulated properly. A pectate lyase (Os04g05050; target of TalAB) is a candidate target gene involved in cell wall degradation. *Xoo* has a range of type II secreted cell wall degrading enzymes including cellulases, a xylanase, a polygalacturonase and an esterase (Tayi et al., 2016a,b). It might, therefore, seem unnecessary to induce host genes involved in cell wall degradation, but it was recently shown that *Pseudomonas syringae* virulence depends on induced expression of an *A. thaliana* polygalacturonase (Wang et al., 2017). Additionally, the *Xanthomonas gardneri*-mediated induction of a pectate lyase in tomato was recently shown to promote disease symptom formation (Schwartz et al., 2017), thus showing that induced pectin degradation in plants is a common strategy for pathogens colonizing plants (Bacete et al., 2018).

Additionally, genes that regulate cell wall composition are potential target genes. The wall-associated kinase (WAK) receptor-like protein OsWAK51 (Os04g43730; target of TalES) is part of a family of pectate receptors. WAKs bind to pectin in the cell wall as well as to pectate fragments, the oligogalacturonic acids and initiate downstream signaling (Kohorn and Kohorn, 2012). They are important for cell expansion and stress responses upon cell wall degradation (Kohorn and Kohorn, 2012). The WAK family is expanded in rice compared to dicotyledonous plants, suggesting functional diversification (Zhang et al., 2005; de Oliveira et al., 2014). OsWAKs were shown to positively or negatively regulate resistance to rice blast and OsWAK18 was identified as the *Xoo* resistance gene *Xa4* in rice variety IRBB4 (Delteil et al., 2016; Hu et al., 2017).

#### Undercover in Waste Management

The last group of potential TALE target genes is involved in protein ubiquitination and protein degradation. Among these genes is the F-box protein OsFBX109 (Os03g51760; target of TalAD). Because the genus *Xanthomonas* is known to undermine host defense by mimicking plant ubiquitin ligases and F-box proteins with the type III effectors XopL and XopI, respectively, further manipulation of the ubiquitination machinery is plausible (Üstün and Börnke, 2014; Erickson et al., 2018).

### A New Generation of Virulence Phenotypes

Most known susceptibility targets of *X. oryzae* TALEs were identified by changes in lesion length based on presence/absence of the corresponding TALE in the *Xanthomonas* strains. However, in most cases only one or two TALEs of *Xoo* or *Xoc* strains show any influence on lesion length (Cernadas et al., 2014). With the knowledge that a lot of TALEs without obvious growth phenotype belong to a highly conserved core TALE class, it seems unlikely that none of them are important for infection. Instead, it is conceivable that these TALEs are benefitting infection without influencing symptom formation and bacterial growth under artificial inoculation conditions. For example, susceptibility factors like PthXo7 or iTALEs/truncTALEs only show their beneficial effect under very specific conditions (Sugio et al., 2007; Ji et al., 2016; Read et al., 2016). In order to identify the virulence function of TALE classes without obvious phenotypes, a targeted approach based on the potential function of the target genes which we have outlined here, will be needed.

## Author Contributions

SM, MR, JG, and JB conceived and designed the experiments. SM, MR, C-AS, SB, JS, and RM performed the experiments. SM, MR, AE, SB, JS, RM, GW, JG, and JB analyzed the data. SM, JG, and JB wrote the manuscript.

## Conflict of Interest Statement

GW and RM are employees of New England Biolabs Inc., Ipswich, MA, United States. The remaining authors declare that the research was conducted in the absence of any commercial or financial relationships that could be construed as a potential conflict of interest.

## Footnotes

1 https://www.ncbi.nlm.nih.gov

2 http://rice.plantbiology.msu.edu/pub/data/Eukaryotic_Projects/o_sativa/annotation_dbs/pseudomolecules/version_7.0/all.dir/all.chrs.con

3 http://rice.plantbiology.msu.edu/pub/data/Eukaryotic_Projects/o_sativa/annotation_dbs/pseudomolecules/version_7.0/all.dir/all.gff3

4 http://rice.plantbiology.msu.edu/pub/data/Eukaryotic_Projects/o_sativa/annotation_dbs/pseudomolecules/version_7.0/all.dir/all.cdna

5 https://www.ebi.ac.uk/ena

6 http://www.jstacs.de/index.php/AnnoTALE
