# Supplementary material for: Transcriptional Reprogramming of Rice Cells by Xanthomonas oryzae TALEs
Source: Front Plant Sci. 2019 Feb 25;10:162. doi: 10.3389/fpls.2019.00162 (PMC6397873; doi:10.3389/fpls.2019.00162)
Supplement: Supplementary file 1 [file Data_Sheet_1.docx]

Supplementary Material

Transcriptional reprogramming of rice cells by
*Xanthomonas oryzae* TALEs

Stefanie Mücke^1^, Maik Reschke^1^, Annett Erkes^2^, Claudia-Alice Schwietzer^1^, Sebastian Becker^1^, Jana Streubel^1^, Richard D. Morgan^3^, Geoffrey G. Wilson^3^, Jan Grau^2^, and Jens Boch^1*^

*** Correspondence:**Jens Boch
[jens.boch@genetik.uni-hannover.de](mailto:jens.boch@genetik.uni-hannover.de)

Supplementary Table 1: Statistics of RNA-seq data.

| Strain | Replicate | Number of raw reads | Number of processed reads | Mapping rate (TopHat) |
| --- | --- | --- | --- | --- |
| PXO142 | 1 | 32094113 | 27957711 | 96.9% |
| PXO142 | 2 | 63611375 | 48448871 | 97.2% |
| PXO142 | 3 | 55959705 | 43864161 | 97.0% |
| ICMP3125 | 1 | 20629128 | 18003212 | 96.9% |
| ICMP3125 | 2 | 45909631 | 34811569 | 97.1% |
| ICMP3125 | 3 | 34444369 | 26844730 | 96.8% |
| MgCl2 | 1 | 39226886 | 34376841 | 97.1% |
| MgCl2 | 2 | 67539256 | 51645048 | 97.2% |
| MgCl2 | 3 | 60838646 | 47755424 | 97.1% |

Number of raw reads, number of reads after processing and quality filtering, and mapping rates reported by TopHat for each of the triplicate experiments after inoculation with *Xoo* PXO142, *Xoo* ICMP3125 and MgCl_2_ as mock, respectively.

Supplementary Table 2: List of potential TALE target genes in rice cultivar Nipponbare.

| Locus ID^1^ | Annotated function | Log2fold change RNAseq | Strain | TALE^2^ | Prediction rank | Position TALE box (bp) |
| --- | --- | --- | --- | --- | --- | --- |
| **LOC_Os01g40290** | expressed protein | 1,894 | ICMP 3125^T^ | TalAA15 | 1 | 268 |
| **LOC_Os01g40290** | expressed protein | 0,887 | PXO142 | TalAA16^X^ | 1 | 268 |
| LOC_Os11g30360 | expressed protein | 0,921 | PXO142 | TalAA16^x^ | 69 | 145 |
| LOC_Os09g30250 | OsSub58 – putative subtilisin homologue | 0,645 | ICMP 3125^T^ | TalAA15 | 2 | 157 |
| LOC_Os04g05050 | pectate lyase precursor | 2,221 | ICMP 3125^T^ | TalAB16 | 49 | 178 |
| LOC_Os10g02840 | O-methyltransferase | 0,638 | ICMP 3125^T^ | TalAB16 | 80 | 107 |
| **LOC_Os03g51760** | OsFBX109 - F-box domain containing protein | 2,734 | ICMP 3125^T^ | TalAD22 | 16 | 101 |
| **LOC_Os03g51760** | OsFBX109 - F-box domain containing protein | 1,368 | PXO142 | TalAD23 | 37 | 101 |
| LOC_Os07g31250 | OsWAK69 - OsWAK receptor-like cytoplasmic kinase OsWAK-RLCK | 0,606 | PXO142 | TalAD23 | 39 | 240 |
| **LOC_Os11g26790** | dehydrin | 1,695 | ICMP 3125^T^ | TalAH11 | 1 | 455 |
| **LOC_Os11g26790** | dehydrin | 1,087 | PXO142 | TalAH12 | 30 | 455 |
| LOC_Os03g40610 | cytochrome P450 | 0,600 | PXO142 | TalAH12 | 1 | 142 |
| LOC_Os02g05390 | retrotransposon protein | 0,673 | PXO142 | TalAL12 | 64 | 235 |
| **LOC_Os06g29790** | phosphate transporter 1 | 1,902 | ICMP 3125^T^ | TalAO15 | 42 | 31 |
| **LOC_Os06g29790** | phosphate transporter 1 | 0,833 | PXO142 | TalAO16 | 2 | 31 |
| LOC_Os02g40410 | expressed protein | 1,657 | ICMP 3125^T^ | TalAO15 | 21 | 117 |
| **LOC_Os07g06970** | HEN1 | 0,824 | PXO142 | TalAP15 | 1 | 301 |
| **LOC_Os07g06970** | HEN1 | 0,687 | ICMP 3125^T^ | TalAP14 | 1 | 301 |
| LOC_Os09g24590 | expressed protein | 0,640 | ICMP 3125^T^ | TalAP14 | 80 | 180 |
| LOC_Os03g03034 | flavonol synthase/flavanone 3-hydroxylase | 1,295 | ICMP 3125^T^ | TalAQ14 | 26 | 315 |
| **LOC_Os09g29820** | bZIP transcription factor domain containing protein | 2,819 | ICMP 3125^T^ | TalAR13 | 2 | 269 |
| **LOC_Os09g29820** | bZIP transcription factor domain containing protein | 2,272 | PXO142 | TalAR14 | 1 | 269 |
| LOC_Os03g61070 | expressed protein | 1,283 | ICMP 3125^T^ | TalAR13 | 70 | 182 |
| LOC_Os11g30360 | expressed protein | 0,921 | PXO142 | TalAR14 | 92 | 16 |
| LOC_Os10g28240 | calcium-transporting ATPase, plasma membrane-type | 0,918 | ICMP 3125^T^ | TalAR13 | 12 | 248 |
| LOC_Os03g62830 | nuclear antigen | 0,894 | PXO142 | TalAS12 | 33 | 389 |
| LOC_Os02g06670 | retrotransposon protein | 3,815 | ICMP 3125^T^ | TalBA8 | 1 | 263 |
| LOC_Os11g42950 | expressed protein | 2,917 | ICMP 3125^T^ | TalBA8 | 68 | 261 |
| LOC_Os07g09020 | argonaute | 1,781 | ICMP 3125^T^ | TalBA8 | 39 | 267 |
| LOC_Os09g07460 | kelch repeat protein | 0,746 | ICMP 3125^T^ | TalBA8 | 27 | 311 |
| LOC_Os03g55530 | HLS | 0,684 | ICMP 3125^T^ | TalBA8 | 3 | 312 |
| LOC_Os02g49350 | plastocyanin-like domain containing protein | 5,163 | PXO142 | TalBH2 | 18 | 269 |
| LOC_Os03g09150 | pumilio-family RNA binding repeat domain containing protein | 2,530 | PXO142 | TalBH2 | 5 | 266 |
| LOC_Os11g31190 | nodulin MtN3 family protein | 2,514 | PXO142 | TalBH2 | 1 | 267 |
| LOC_Os01g19330 | MYB family transcription factor | 0,950 | PXO142 | TalBH2 | 22 | 265 |
| LOC_Os04g43800 | phenylalanine ammonia-lyase | 0,735 | PXO142 | TalBK2 | 49 | 268 |
| LOC_Os08g04800 | triacylglycerol lipase like protein | 1,898 | ICMP 3125^T^ | TalBM2 | 66 | 245 |
| LOC_Os01g73890 | transcription initiation factor IIA gamma chain | 1,079 | ICMP 3125^T^ | TalBM2 | 2 | 270 |
| LOC_Os04g49970 | U-box | 0,973 | ICMP 3125^T^ | TalBM2 | 67 | 259 |
| LOC_Os06g49860 | methyltransferase | 0,899 | ICMP 3125^T^ | TalBM2 | 55 | 248 |
| LOC_Os06g09350 | expressed protein | 0,833 | ICMP 3125^T^ | TalBM2 | 74 | 239 |
| LOC_Os04g43730 | OsWAK51 - OsWAK receptor-like protein kinase | 5,762 | ICMP 3125^T^ | TalES1 | 60 | 279 |
| LOC_Os06g03710 | DELLA protein SLR1 | 1,591 | ICMP 3125^T^ | TalES1 | 34 | 447 |

^1^ potential target genes induced by both strains are shown in bold

^2^ TALEs without a functional activation domain and therefore impaired transcription activation are marked with a superscript x.

Supplementary Table 3: RVD composition of TALEs in PXO142 and ICMP 3125^T^.

| TALE | repeat number | RVD sequence^1^ | *Xoo* strain of origin |
| --- | --- | --- | --- |
| AA15 | 19.5 | NI HG NS HG HG HD NS NG HD NN NG HG NG HD HG HD HD NI NN NG | ICMP 3125^T^ |
| AB16 | 17.5 | NI HG NI NI NI NN HD NS NN NS NN HD NN NI HD NN NS NG | ICMP 3125^T^ |
| AB17 | 19.5 | NI HG NI NI NI NN HD NS NN NS NN HD NN NI HD NN NI NG HD NG | PXO142 |
| AD22 | 23.5 | NN HD NS NG HD NN N* NI HD NN HD NN HD NN HD NN NN NN NN NN NN NN HD NG | ICMP 3125^T^ |
| AD23 | 23.5 | NN HD NS NG HD NN N* NI HD NS HD NN HD NN HD NN NN NN NN NN NN NN HD NG | PXO142 |
| AE15 | 12.5 | NI NN NI HG HG NV HG HD HG HD HD HD NG | ICMP 3125^T^ |
| AE16 | 12.5 | NI NN NI HG HG HD NG HD HG HD HD HD NG | PXO142 |
| AF17 | 15.5 | NI NN NN NI NI NI HD NS HG NN NN NN NI NI HG HD | ICMP 3125^T^ |
| AF18 | 15.5 | NI NN NN NI NI NI HD NS HG NN NN NN NI NI NG HD | PXO142 |
| AG15 | 19.5 | NI NG NN NG NK NG NI NN NI NN NI NN NS NG NS NN NI N* NS NG | PXO142 |
| AH11 | 19.5 | NI N* NI NS NN NG NN NS N* NS NN HD N* NI HG HD NI HD HD NG | ICMP 3125^T^ |
| AH12 | 19.5 | NI N* NI NS NN NG NN NS N* NS NN NS N* HD HG HD NI HD HD NG | PXO142 |
| AL11 | 17.5 | NI NS HD NG NS NN HD N* NN NN NI NG HD NG HD HD HD NG | PXO142 |
| AL12 | 17.5 | NI NS HD NG NS NN HD N* NN NN NS NN HD HG HD HD NN NG | PXO142 |
| AN14 | 20.5 | NI HG NI HG NI NI NI HD NN HD NS NG SS HD NI NI NN NI NN NI NG | ICMP 3125^T^ |
| AN15 | 20.5 | NI N* NI HG NI NI NS HD NN HD NS NG SS HD NI NI NN NI NN NI NG | PXO142 |
| AO15 | 16.5 | NI NN N* NG NS NN NN NN NI NN NI N* HD HD NI NG NG | ICMP 3125^T^ |
| AO16 | 16.5 | NI NN N* NG NS NN NN NN NI NN NI N* HD HD NI NG NG | PXO142 |
| AP14 | 19.5 | HD HD HD NG N* NN HD HD N* NI NI NN NN HI ND HD NI HD NG NG | ICMP 3125^T^ |
| AP15 | 19.5 | HD HD HD NG N* NN HD HD N* NI NI NN HD HI ND HD NI HD NG NG | PXO142 |
| AQ14 | 26.5 | HD HD NN NN NG NG HD NS HG HD NG N* HD HD HD N* NN NI NN HD HI ND HD HG NN HG N* | ICMP 3125^T^ |
| AR13 | 22.5 | NI H* NI NN NN NN NN NN HD NI HD HG HD NI N* NS NI NI HG HD NS NS NG | ICMP 3125^T^ |
| AR14 | 22.5 | NI H* NI NN NN NN NN NN HD NI NS HG HD NI N* NS NI NI HD HD N* NS N* | PXO142 |
| AS12 | 26.5 | NI HG NI NI HG HD NN HD HD HD NI NI NN NI HD HD HD HG NN NN HD NS NN HD NG NS N* | PXO142 |
| BA8 | 15.5 | NI NS HD HG NS NN HD H* NG NN NN HD HD NG HD NG | ICMP 3125^T^ |
| BH2 | 28.5 | NI HG NI HG NI NI NI HD NN HD HD HD NG HD N* NI HD HD NN NS NI NN NN NG NN HD N* NS N* | PXO142 |
| BK2 | 21.5 | NI HG NI NN NI NN HD NI HD HD NS NS HD NI NI HD NG HD HD HD NG NG | PXO142 |
| BM2 | 21.5 | NI NG NI NI N* NN HD HD N* NI NI NI NG HD HG NN NS NN HD HD NG N* | ICMP 3125^T^ |
| ES1 | 11.5 | NN HD NI HG HD NG N* HD NI N* NS N* | ICMP 3125^T^ |
| ET1 | 33.5 | NI H* NN HD H* NG NN NN HD HD NG HD NI HD HG NS NI HG N* NN HD NI NG HG NN NN HD NS NN HD N* NI NI N* | ICMP 3125^T^ |

^1^TalAQ14, TalAS12, TalBH2 and TalBK2 have repeats with 42, 40, 39 and 36 amino acids, respectively, that are underlined.

Supplementary Table 4: qRT-PCR data used in Figure 4.

| Gene | log(10) fold change ± standard deviation | | | | |
| --- | --- | --- | --- | --- | --- |
|  | PXO83 | PXO142 | ICMP 3125^T^ | Roth X1-8 | Roth X1-8 +TALE |
| *OsSWEET14* | 3,54±0,35 | 3,32±0,08 | 0,14±0,04 | 0,54±0,07 | 3,50±0,16 |
| *OsPHO1;3* | 0,52±0,28 | 0,80±0,06 | 1,23±0,37 | -0,16±0,37 | 0,74±0,07 |
| *OsNPF6.3* | 0,40±0,04 | 0,35±0,01 | 0,54±0,05 | 0,15±0,13 | 0,87±0,03 |
| *OsLsi1* | 0,25±0,07 | 0,85±0,01 | 0,13±0,13 | 0,14±0,04 | 0,66±0,12 |
| *OsHLS1* | 0,44±0,15 | -0,04±0,11 | 0,45±0,18 | 0,05±0,10 | 0,86±0,01 |
| *OsDOX-1* | 1,27±0,10 | 1,04±0,07 | 1,60±0,22 | 0,06±0,04 | 2,22±0,04 |
| *OsTFIIAγ1* | 0,05±0,06 | 0,01±0,04 | 1,44±0,03 | -0,04±0,08 | 0,46±0,06 |
| *OsTFX1* | 1,84±0,16 | 1,81±0,14 | 1,72±0,09 | -0,11±0,08 | 1,68±0,08 |
| *OsHEN1* | 1,24±0,04 | 1,13±0,04 | 0,96±0,03 | -0,10±0,03 | 1,84±0,10 |
| *OsPL* | 1,96±0,10 | 1,87±0,05 | 2,37±0,01 | -0,21±0,07 | 3,07±0,04 |
| *OsWAK51* | 0,80±0,18 | 0,25±0,22 | 4,15±0,14 | 0,45±0,19 | 4,44±0,32 |
| *OsFBX109* | 0,96±0,12 | 0,67±0,07 | 1,19±0,01 | -0,07±0,33 | 0,72±0,09 |

Supplementary Table 5: RVD sequences of TalAL members in PXO142.

| TALE | repeat number | RVD sequence^1^ | *Xoo* strain of origin |
| --- | --- | --- | --- |
| AL11 | 17,5 | NI NS HD NG NS NN HD N* NN NN **NI** **NG** HD **NG** HD HD **HD** NG | PXO142 |
| AL12 | 17,5 | NI NS HD NG NS NN HD N* NN NN **NS** **NN** HD **HG** HD HD **NN** NG | PXO142 |

^1^ Differences in RVD sequences are shown in bold.

Supplementary Table 6: Used oligonucleotides.

| oligo name | sequence | description |
| --- | --- | --- |
| lacIII-E-F-SM | tttgcaatgcaGCCAGGCGCATGGCCttacaagtc | cloning |
| lacIII-E-R-SM | tttgcaatgtcaTGGCACAGCACCGGCAACAGCCGC | cloning |
| lacIII-F-F-SM | tttgcaatgcaGCGGCTGTTGCCGGTGCTGTGCCAGGCGCATGGCC | cloning |
| lacIII-F-R-SM | tttgcaatgtcaCCGCTGCACCGTCTCCAAAGTctt | cloning |
| pSKX1_EV_F/SM | TATGGCAGGAGCT | cloning |
| pSKX1_EV_R/SM | CACCAGCTCCTGC | cloning |
| pSKX1_GFP_F/SM | TTTGGTCTCTTATGGTGAGCAAGGGCGAGG | cloning |
| pSKX1_GFP_R/SM | TTTGGTCTCTCACCCATATGCTTGTACAGCTCGTCCA | cloning |
| 1000bpFlavo_F | TTTGGTCTCACACCCTCTATGCGACATCCTATATAG | cloning |
| 1000bpFlavo_R | TTTGGTCTCACCTTGCCGCGGTACACACACACAAC | cloning |
| 1000bpPhos_F | TTTGGTCTCACACCTAGGACAAGTAGTACTACTC | cloning |
| 1000bpPhos_R | TTTGGTCTCACCTTGGGGAGAGGTGTACTTATACG | cloning |
| P_Aquap_F/SM | TTTGGTCTCTCACCCTTATTGTAATGTCACCTTTGCAAA | cloning |
| P_Aquap_R/SM | TTTGGTCTCTCCTTTTCTGACGCTCTATCTAGCTG | cloning |
| P_HEN1_F/SM | TTTGGTCTCTCACCATTTTATTGGATGCATGCATTGTATTTA | cloning |
| P_HEN1_R/SM | TTTGGTCTCTCCTTCAAACGCCCAAAAAAAACAAACAAAA | cloning |
| P_HLS_F/SM | TTTGGTCTCTCACCCGCCATACCAATATTTTAGCGTTA | cloning |
| P_HLS_R/SM | TTTGGTCTCTCCTTGTATATATGCCCGGTGATTGG | cloning |
| P_OsFBX109neuF/SM | TTTGGTCTCTCACCGTGCCACACACCCTCTAC | cloning |
| P_OsFBX109neuR/SM | TTTGGTCTCTCCTTAGCCGGACCACCGACAAC | cloning |
| P_OsPTR2neuF/SM | TTTGGTCTCTCACCCTACAGTAAACTCAATTCCATCTAT | cloning |
| P_OsPTR2neuR/SM | TTTGGTCTCTCCTTCTTCTCTCTCTCTCTTCTTCTTC | cloning |
| P_PectLy_F/SM | TTTGGTCTCTCACCAAAAATACAGTAATTAGTTGCAGGACA | cloning |
| P_PectLy_R/SM | TTTGGTCTCTCCTTTGCTCCCGCGCCTCGAC | cloning |
| P_TFIIA_F/SM | TTTGGTCTCTCACCGAATGATAAACTTTAATAGTTTAATTTGC | cloning |
| P_TFIIA_R/SM | TTTGGTCTCTCCTTCGATGATCGAATATCGATCCC | cloning |
| P_TFX1_F/SM | TTTGGTCTCTCACCATTATAGGCTTATAGAAGCACACC | cloning |
| P_TFX1_R/SM | TTTGGTCTCTCCTTGGCTGTTTTCGCTTGCTTAGT | cloning |
| P_WAK51_F/SM | TTTGGTCTCTCACCTGAAACTTGAGGGACTAAATTAACTA | cloning |
| P_WAK51_R/SM | TTTGGTCTCTCCTTGCCAGTATATATGGAGATGTATTG | cloning |
| P_Os04g49194_F | TTTGGTCTCACACCGACTTTATGAATCTTAATATATG | cloning |
| P_Os04g49194_R | TTTGGTCTCACCTTGGGAGACGAAGCGCGAG | cloning |
| 11N3_alternativ_F2/JS | CTACCTGGCCCCACTGC | qRT-PCR |
| 11N3_alternativ_R/JS | GTGCGCACCACCAGCC | qRT-PCR |
| Os03g03034_F3 | CACGGCTTCTTCCAGGTGCTCA | qRT-PCR |
| Os03g03034_R3 | TTCTGTCTTCCTGTGATACCAGCACT | qRT-PCR |
| Os04g05050_F3 | CTCTCCAACTGCGCCGACGG | qRT-PCR |
| Os04g05050_R3 | GCGGTTGCCCTGGCTGTTGA | qRT-PCR |
| Os06g29790_F3 | TCCTCCACGGAGATCAGTCCCT | qRT-PCR |
| Os06g29790_R3 | ACAGAAGCTCACGGATGGCGG | qRT-PCR |
| OsHen1_qrt_F/MR | TATGCCAGACCAATGCTGAAGTG | qRT-PCR |
| OsHen1_qrt_R/MR | GATTGCCCTCGACAAGCTTGG | qRT-PCR |
| OsTFIIAy_qRT_F/MR | GCCACCTTCGAGCTGTACCG | qRT-PCR |
| OsTFIIAy_qRT_R/MR | TACTCTTCTTTAGTCTCCAGCAATTTG | qRT-PCR |
| OsTFX1_qRT-F/MR | TTACCATGGCGAGGTGGCC | qRT-PCR |
| OsTFX1_qRT-R1/MR | CGGCCCTCTCCTTCCTGAG | qRT-PCR |
| qRT_Aquap_F1/SM | CATCGCCGACTTCTTCCCTC | qRT-PCR |
| qRT_Aquap_R1/SM | ATATCGCTCCGGTGAACTGC | qRT-PCR |
| qRT_FBX109_F4/SM | TGGATCGGCAAGACACACGA | qRT-PCR |
| qRT_FBX109_R4/SM | GTCGCGAGGTCGAGGGATAC | qRT-PCR |
| qRT_HLS_F2/SM | GGCAATGGCAGGGAGATCAT | qRT-PCR |
| qRT_HLS_R2/SM | GTCCGGAACTTGGAGTAGCC | qRT-PCR |
| qRT_PTR2_F3/SM | GATCGCCGTGAACCTGGTCA | qRT-PCR |
| qRT_PTR2_R3/SM | TAGAGCGCCAGGTACAGCAC | qRT-PCR |
| qRT_WAK51_F2/SM | GCTCTCATGGATACGAAGGTAACC | qRT-PCR |
| qRT_WAK51_R2/SM | ATACCGTTCCATGACCTCCTTGG | qRT-PCR |

Supplementary Figure 1: Coverage plot of the assembled chromosome of *Xoo* ICMP3125.

Coverage values are those reported by the Resequencing.1 pipeline of the Pacific Biosciences SMRT Portal. Positions of predicted TALE genes are indicated by vertical blue lines (solid: forward strand; dashed: reverse complementary strand).

Supplementary Figure 2: Coverage plot of the assembled chromosome of *Xoo* PXO142.

Coverage values are those reported by the Resequencing.1 pipeline of the Pacific Biosciences SMRT Portal. Positions of predicted TALE genes are indicated by vertical blue lines (solid: forward strand; dashed: reverse complementary strand).

Supplementary Figure 3: Phylogenetic tree of fully sequenced Asian *Xoo* strains.

The available fully sequenced Asian *Xoo* strains were used to create a phylogenetic tree based on conserved genes. The African *Xoo* strain AXO1947 was used as an outgroup. The color scale of “Subtree Similarity” indicates the Jaccard index between sets of subtree leaves.


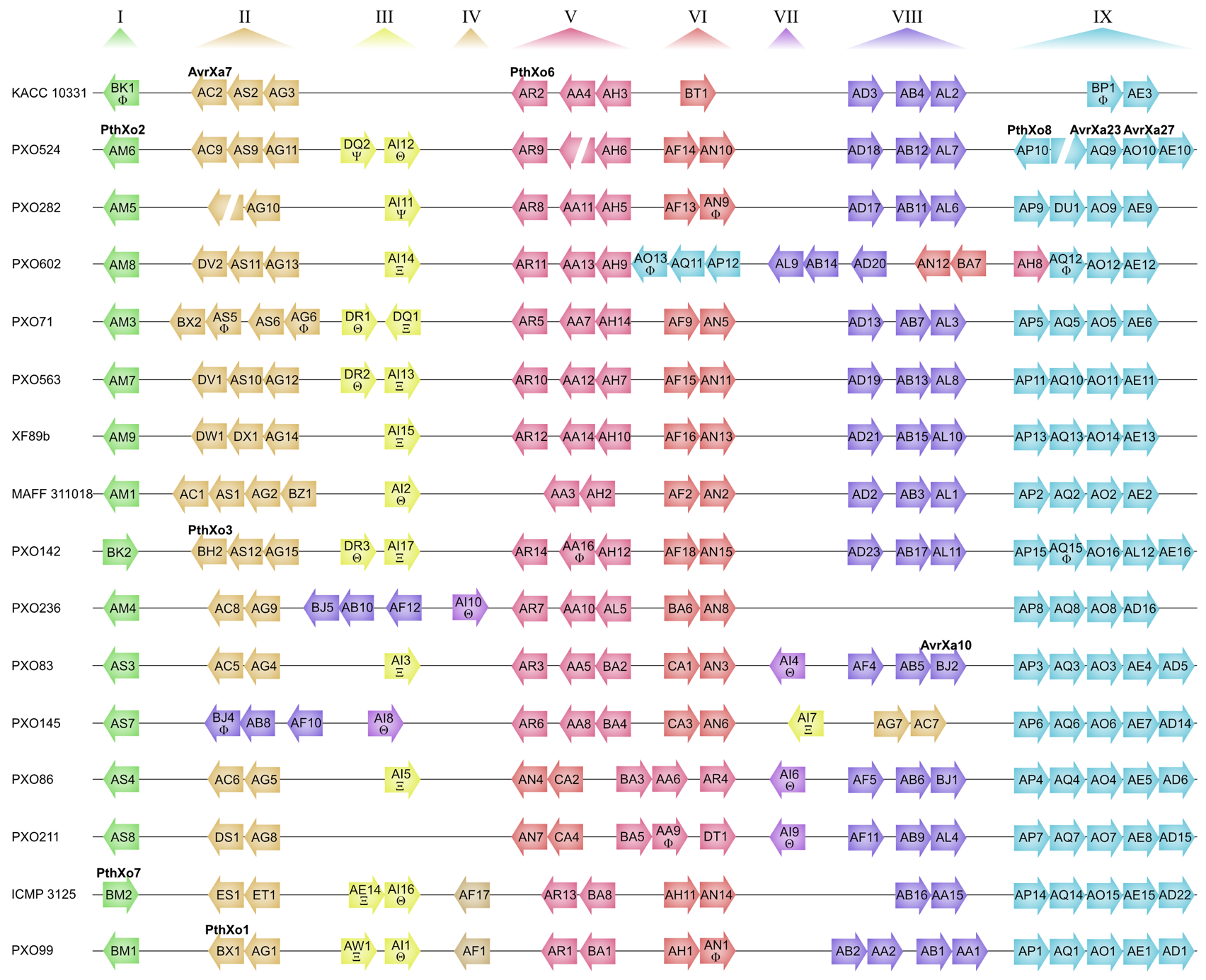


Supplementary Figure 4: Overview of TALE clusters for all sequenced Asian *Xoo* strains.

*TALE* genes are represented by arrows indicating their relative orientation in the genome. All TALEs are assigned into classes by AnnoTALE and named accordingly, while the alternative name of prominent members of the classes are indicated in bold. Truncated TALEs (truncTALEs/ iTALEs) are labelled with xi (Ξ) for truncTALE-related/iTALE type A or theta (Θ) for truncTALE/iTALE type B. Other *TALE* pseudogenes without functional N- or C-terminal regions are marked with a phi (Φ). Previously established TALE clusters are specified at the top and cluster affiliation of individual *TALE* genes is shown by color.


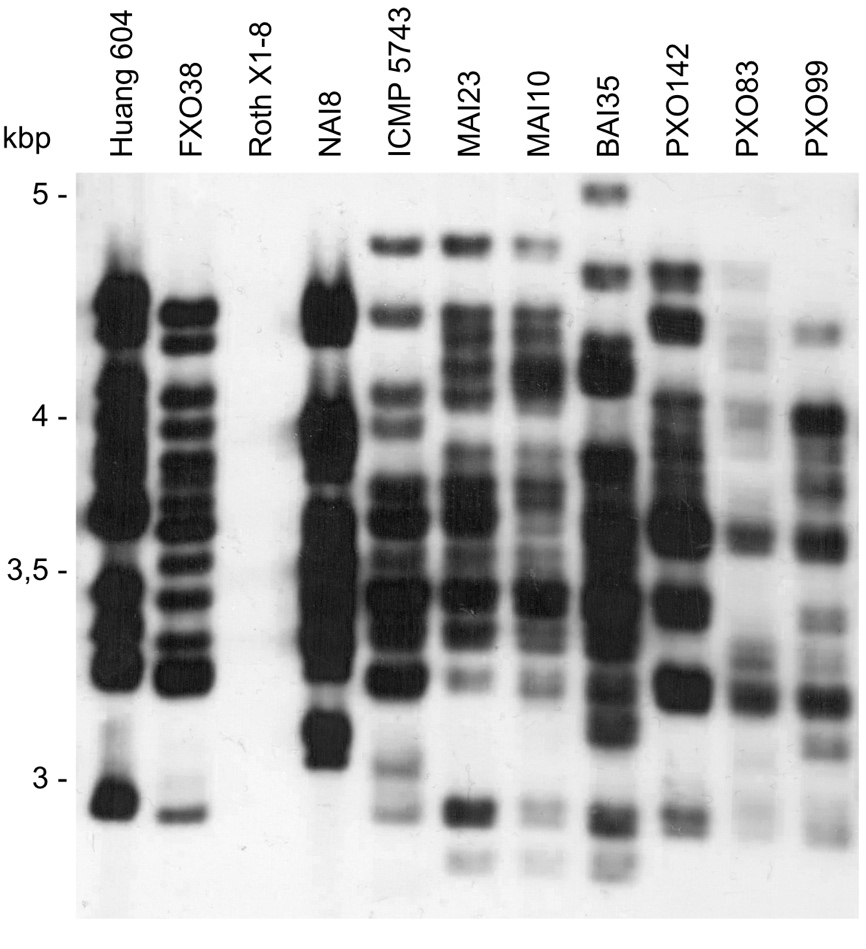


Supplementary Figure 5: Southern Blot analysis of *Xanthomonas oryzae* strains.

Genomic DNA of different *Xoo* and *Xoc* strains was digested with *Bam*HI, separated on an agarose gel and transferred to a nylon membrane. *TALE* gene-containing fragments were detected with a DIG-labelled probe corresponding to 500 bp of the 3’ part of *talC*.


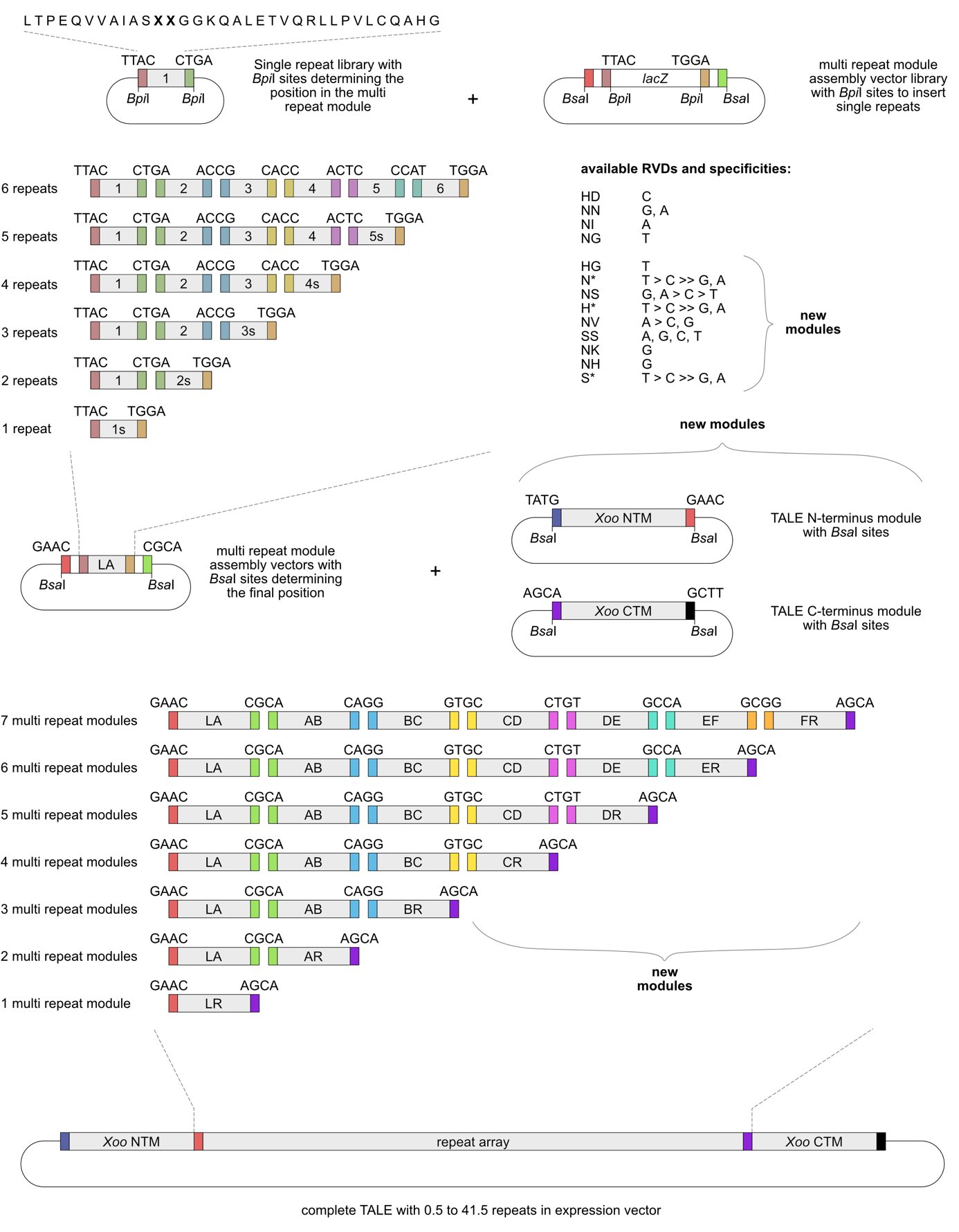


Supplementary Figure 6: Overview of Golden TALE Cloning Kit with new modules.

Single repeat modules have flanking *Bpi*I sites determining their position in the multi repeat modules assembled in the first cloning step. Available single repeat modules are listed with their RVDs and corresponding DNA binding specificities. In order to create multi repeat modules with less than six repeats, “stop repeats” (1s - 5s) can be employed. Up to seven multi repeat modules can be combined with the TALE N-terminal region (NTM) and the C-terminal region (CTM) in the second cloning step using their flanking *Bsa*I sites.

ATGATGGATCCCATTCGTTCGCGCACGCCAAGTCCTGCCCGCGAGCTTCTGCCCGGACCCCAACCGGATAGGGTTCAGCCGACTGCAGATCGGGGGGGGGCTCCGCCTGCTGGCGGCCCCCTGGATGGCTTGCCCGCTCGGCGGACGATGTCCCGGACCCGGCTGCCATCTCCCCCTGCGCCCTCGCCTGCGTTCTCGGCGGGCAGCTTCAGCGATCTGCTCCGTCAGTTCGATCCGTCGCTTCTTGATACATCGCTTCTTGATTCGATGCCTGCCGTCGGCACGCCGCATACAGCGGCTGCCCCAGCAGAGTGCGATGAGGTGCAATCGGGTCTGCGTGCAGCCGATGACCCGCCACCCACCGTGCGTGTCGCTGTCACTGCCGCGCGGCCGCCGCGCGCCAAGCCGGCCCCGCGACGGCGTGCGGCGCAACCCTCCGACGCTTCGCCGGCCGCGCAGGTGGATCTACGCACGCTCGGCTACAGTCAGCAGCAGCAAGAGAAGATCAAACCGAAGGTGCGTTCGACAGTGGCGCAGCACCACGAGGCACTGGTGGGCCATGGGTTTACACACGCGCACATCGTTGCGCTCAGCCAACACCCGGCAGCGTTAGGGACCGTTGCTGTCACGTATCAGGACATAATCACGGCGTTGCCAGAGGCGACACACGAAGACATCGTTGGCGTCGGCAAACAGTGGTCCGGCGCACGCGCCCTGGAGGCCTTGCTCACGGAGGCGAGGGAGTTGAGAGGTCCGCCGTTACAGTTGGACACAGGCCAACTTCTCAAGATTGCAAAACGTGGCGGCGTGACCGCAGTGGAGGCAGTGCATGCATGGCGCAATGCACTGACGGGTGCCCCCCTGAAC

Supplementary Figure 7: TALE N-terminal region from TalAG4 of *Xoo* strain PXO83.

AGCAAGCATTGTTGCCCAGTTATCTCGCCCTGATCCGGCGTTGGCCGCGTTGACCAACGACCACCTCGTCGCCTTGGCCTGCCTCGGCGGACGTCCTGCCCTGGATGCAGTGAAAAAGGGATTGCCGCACGCGCCGGAATTGATCAGAAGAGTCAATAGCCGTATTGGCGAACGCACGTCCCATCGCGTTGCCGACTACGCGCAAGTGGTTCGCGTGCTGGAGTTTTTCCAGTGCCACTCCCACCCAGCGTACGCATTTGATGAGGCCATGACGCAGTTCGGGATGAGCAGGCACGGGTTGGTACAGCTCTTTCGCAGAGTGGGCGTCACCGAATTCGAAGCCCGCTGCGGAACGCTCCCCCCAGCCTCGCAGCGTTGGGACCGTATCCTCCAGGCATCAGGGATGAAAAGGGCCAAACCGTCCCCTACTTCAGCTCAAACGCCGGATCAGGCGTCTTTGCATGCATTCGCCGATTCGCTGGAGCGTGACCTTGATGCGCCCAGCCCAATGCACGAGGGAGATCAGACGCGGGCAAGCAGCCGTAAACGGTCCCGATCGGATCGTGCTGTCACCGGCCCCTCCGCACAGCAATCTTTCGAGGTGCGCGTTCCCGAACAGCGCGATGCGCTGCATTTGCCCCTCAGCTGGAGGGTAAAACGCCCGCGTACCAGGATCGGGGGCGGCCTCCCGGATCCTGGTACGCCCATCGCTGCCGACCTGGCAGCGTCCAGCACCGTGATGTGGGAACAAGATGCGGCCCCCTTCGCAGGGGCAGCGGATGATTTCCCGGCATTCAACGAAGAGGAGCTCGCATGGTTGATGGAGCTATTGCCTCAGTCAGGCTCAGTCGGAGGGACGATCGGTG

Supplementary Figure 8: TALE C-terminal region from TalAO3 of *Xoo* strain PXO83.


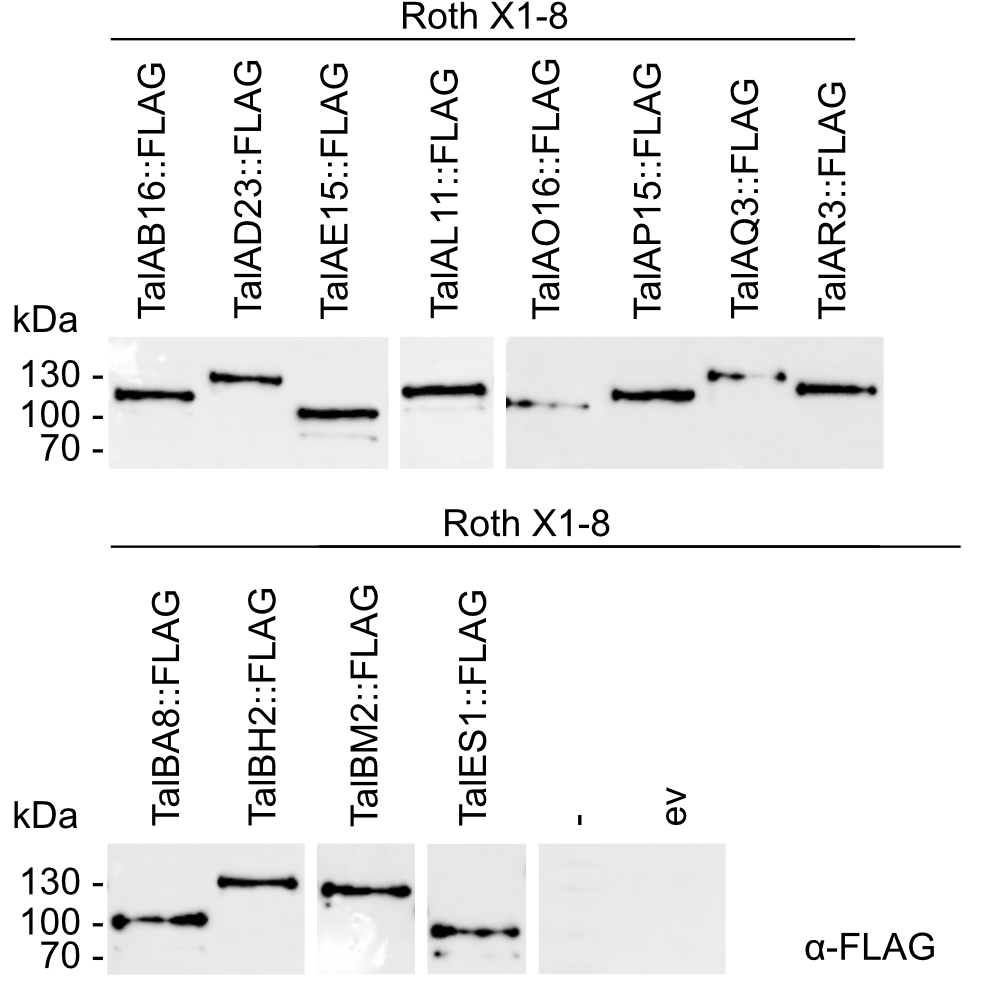


Supplementary Figure 9: Western blot analysis of Roth X1-8 strains with artificial TALEs.

Derivatives of the *Xoo* strain Roth X1-8 containing TALE::FLAG expression constructs were harvested from liquid culture, the whole protein content was separated on SDS gels and transferred on nitrocellulose membranes. Protein synthesis of TALEs was detected using α-FLAG primary antibody from mouse and α-mouse secondary antibody coupled with horseradish peroxidase. ev: empty vector, -: without plasmid


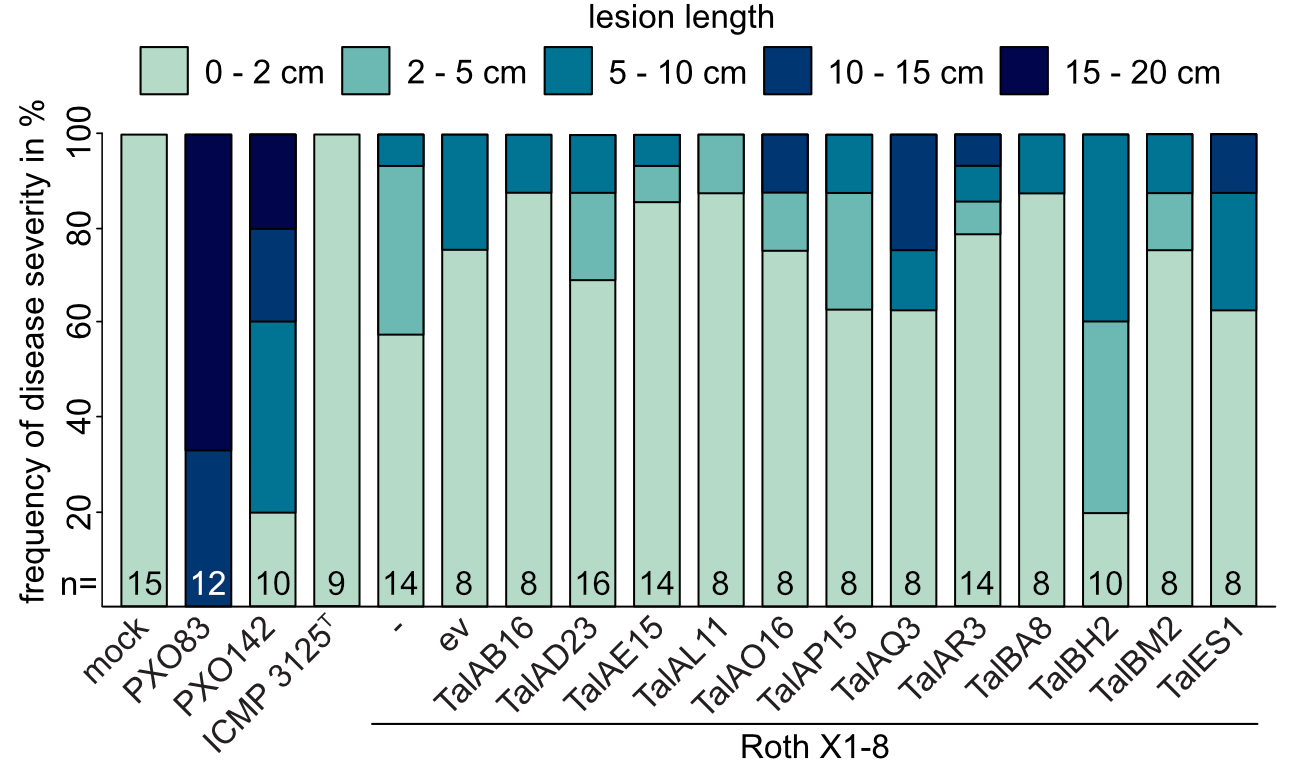


Supplementary Figure 10: Virulence assay of *Xoo* strains infecting rice.

The rice cultivar Nipponbare was infected with *Xoo* strains PXO83, PXO142, ICMP 3125^T^ and Roth X1-8 as well as Roth X1-8 containing single TALE expression constructs. Infection was done by clipping the third leaf with bacterial solution. 14 days after infection leaves were harvested and lesion length was measured. Disease severity was scored in five different categories: 0 – 2 cm, 2 – 5 cm, 5 – 10 cm, 10 – 15 cm and 15 – 20 cm. Infections were done in four independent experiments and number of infected plants (n) is shown in the bars. Depicted is the frequency of different disease severity categories observed through all experiments. ev – empty vector


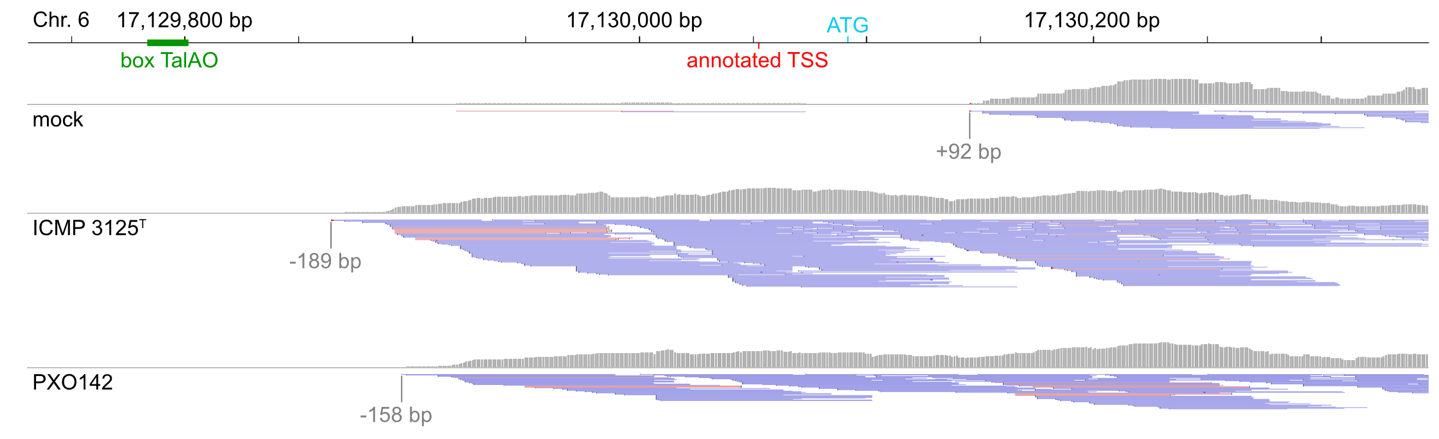


Supplementary Figure 11: TALE-mediated change of transcription start site of *OsPHO1;3*.

RNAseq reads of rice cultivar Nipponbare inoculated with *Xoo* ICMP 3125^T^, PXO142 or a mock control were mapped on the Nipponbare genome. An excerpt of chromosome 6 (Chr. 6) is depicted showing the region around the annotated transcription start site (TSS) of *OsPHO1;3.* The TALE box of the corresponding TALE class TalAO is shown in green and the translational start site is marked as ATG. The transcription start, as shown by RNAseq reads, is labelled relative to the annotated transcription start site.

Supplementary Figure 12: Alignment of AtDMR6 and rice homologs OsDOX-1 and OsDOX-2.

The amino acid sequences of AtDMR6 and the homologs OsDOX-1 (Os03g03034) and OsDOX-2 (Os04g49194) were aligned using the Geneious Alignment tool. Identical residues are marked with black background and similar residues according to Blosum62 are colored grey.

Supplementary Figure 13: Functional convergence on the putative silicone transporter *OsLsi1*.

**(A)** New cases of functional convergence of TALEs are shown schematically for *OsLsi1*. 250 bp upstream of the start codon of target genes are displayed and TALE boxes are highlighted in grey. TALE boxes are magnified and matched to the RVD sequences of the corresponding TALEs. Mismatches are indicated by black dots. Affiliation of TALEs with *Xoo* or *Xoc* is shown on the right next to the TALEs. **(B)** 1000 bp upstream of the ATGs of the TALE target genes were amplified from rice cultivar Nipponbare DNA and cloned in front of a promoterless *uidA* reporter gene. Artificial TALEs were assembled with RVD sequences shown above and Hax3 N- and C-terminal regions under control of a 35S promoter. *A. tumefaciens* strains delivering the reporter constructs and strains delivering the TALE expression constructs were co-inoculated into *N. benthamiana* leaves and β-glucoronidase measurements were performed 2 dpi. Quantitative GUS activity measurements were performed three times with samples obtained as described above. Error bars represent standard deviation between triplicates. The statistical significance between samples with and without corresponding TALEs is indicated by p-values resulting from an unpaired t-test (p<0.1 - *; <0.05 - **; <0.01 - ***). The TALE Hax3 is used as a negative control (-). Histochemical GUS staining of leaf discs and quantitative GUS activity measurements were done in parallel from the same plants. Leaf discs were stained for 4 h in GUS staining solution, destained in 96% ethanol and documented. One representative leaf disc per combination is shown.
